# Chronische Entzündungserkrankungen in Deutschland: Eine Querschnittanalyse über Begleiterkrankungen und Arzneimitteleinsatz

**DOI:** 10.1007/s00393-022-01306-1

**Published:** 2023-01-04

**Authors:** Jan Leipe, Renate Schmelz, Gabriela Riemekasten, Diamant Thaçi, Jörg Henes, Knut Schäkel, Andreas Pinter, Michael Sticherling, Joanna Wegner, Stefano Fusco, Miriam Linke, Valeria Weber, Karina C. Manz, Holger Bartz, Marit Roecken, Sandra Schmidt, Bimba F. Hoyer

**Affiliations:** 1 Sektion Rheumatologie, V. Medizinische Klinik, Universitätsklinikum Mannheim, Theodor-Kutzer-Ufer 1–3, 68167 Mannheim, Deutschland; 2 Medizinische Klinik und Poliklinik I, Universitätsklinikum Carl Gustav Carus Dresden, Dresden, Deutschland; 3 Klinik für Rheumatologie und klinische Immunologie, Universitätsklinikum Schleswig-Holstein, Lübeck, Deutschland; 4 Institut und Exzellenzzentrum für Entzündungsmedizin, Universität zu Lübeck, Schleswig-Holstein, Lübeck, Deutschland; 5 Zentrum für interdisziplinäre und klinische Immunologie, Rheumatologie und autoinflammatorische Erkrankungen (INDIRA) und innere Medizin II, Universitätsklinikum Tübingen, Tübingen, Deutschland; 6 Hautklinik, IZEH – Interdisziplinäres Zentrum für chronisch entzündliche Erkrankungen, Universitätsklinikum Heidelberg, Heidelberg, Deutschland; 7 Klinik für Dermatologie, Venerologie und Allergologie, Universtitätsklinikum Frankfurt, Frankfurt, Deutschland; 8 Hautklinik, Psoriasiszentrum, Deutsches Zentrum für Immuntherapie, Universitätsklinikum Erlangen, Erlangen, Deutschland; 9 Hautklinik und Poliklinik, Universitätsitätsmedizin der Johannes-Gutenberg-Universität Mainz, Mainz, Deutschland; 10 Medizinische Klinik I, Universitätsklinikum Tübingen, Tübingen, Deutschland; 11 Klinik für Dermatologie, Venerologie und Allergologie, Universitätsmedizin Mannheim, Mannheim, Deutschland; 12 IGES Institut GmbH, Berlin, Deutschland; 13 Janssen-Cilag GmbH, Neuss, Deutschland; 14 Klinik für Innere Medizin I, Sektion Rheumatologie und klinische Immunologie, Universitätsklinikum Schleswig-Holstein, Kiel, Deutschland

**Keywords:** Chronische Entzündungserkrankungen, Epidemiologie, Medikamentöse Versorgung, GKV-Routinedatenanalyse, Real-World-Evidenz, Chronic inflammatory diseases, Epidemiology, Pharmacotherapy, Statutory health insurance data analysis, Real-world evidence

## Abstract

**Hintergrund:**

Chronische Entzündungserkrankungen (engl. „immune-mediated inflammatory diseases“ [IMID]) sind mit einer erheblichen Krankheitslast verbunden. Diese ist umso ausgeprägter, sofern diese gleichzeitig bei Patienten auftreten oder z. B. weitere Komorbiditäten bestehen. Die Versorgung der IMID ist komplex und involviert diverse medizinische Fachdisziplinen.

**Ziel der Arbeit:**

Die Beschreibung der Krankheitslast und der aktuellen Arzneimittelversorgung der Patienten mit IMID.

**Material und Methoden:**

Die retrospektive Querschnittanalyse erfolgte unter Nutzung von Abrechnungsdaten der gesetzlichen Krankenversicherung der InGef-Datenbank. Unter 3.988.695 Versicherten wurden im Jahr 2018 anhand dokumentierter Diagnosen (ICD-10-GM) prävalente Patienten mit Psoriasis (Pso), Psoriasisarthritis (PsA), Spondyloarthritiden (SpA), rheumatoider Arthritis (RA), Morbus Crohn (MC), Colitis ulcerosa (CU) oder Kollagenosen identifiziert. Das gemeinsame Auftreten verschiedener IMID sowie weiterer Begleiterkrankungen wurde im Vergleich zur Referenzpopulation untersucht. Die medikamentöse Versorgung wurde basierend auf vordefinierten Therapieformen beschrieben.

**Ergebnisse:**

Im Jahr 2018 wurden 188.440 Patienten mit IMID (4,7 %) identifiziert. Im Vergleich zur Referenzpopulation war die Prävalenz von depressiven Episoden und kardiovaskulären Risikoerkrankungen bei Patienten mit IMID höher. Bei MC, CU, RA, und PsA wurden DMARDs (engl. „disease-modifying antirheumatic drugs“) und klassische systemische Therapien am häufigsten eingesetzt. Bei Pso, SpA und Kollagenosen waren NSAR (nichtsteroidale Antirheumatika) die häufigsten Therapieformen oft in Kombination mit anderen Wirkstoffen.

**Diskussion:**

Ein beträchtlicher Anteil der Patienten mit IMIDs (16,9–27,5 %) leidet an unterschiedlichen Erkrankungen des IMID-Formenkreises. Sie sind häufig von Begleiterkrankungen betroffen und bedürfen einer interdisziplinären medizinischen Versorgung.

**Zusatzmaterial online:**

Die Online-Version dieses Beitrags (10.1007/s00393-022-01306-1) enthält die Tabellen S1–S10.

## Abstract (en)

**Background:**

Immune-mediated inflammatory diseases (IMID) can lead to a substantial disease burden for those affected, in particular by the concomitant occurrence of other IMIDs or in the presence of comorbidities. The care of patients with IMIDs is complex and involves various medical disciplines.

**Objective:**

To describe the burden of disease and the current routine drug treatment of patients with IMID.

**Material and methods:**

The retrospective cross-sectional analysis was based on statutory health insurance claims data from the InGef database. Prevalent patients with psoriasis (Pso), psoriatic arthritis (PsA), spondylarthritis (SpA), rheumatoid arthritis (RA), Crohn’s disease (MC), ulcerative colitis (CU), or connective tissue disease were identified among 3,988,695 insured patients in 2018. The concomitant occurrence of different IMIDs and the extent to which patients with IMID are affected by other comorbidities compared to a reference population were investigated. The current routine drug treatment was described based on the use of predefined forms of treatment.

**Results:**

In the database 188,440 patients with IMID (4.7%) were identified. Compared to the reference population the prevalence of comorbidities, such as depressive episodes and cardiovascular risk factors was higher in patients with IMID. For MC, CU, RA, and PsA disease-modifying antirheumatic drugs (DMARD) and classical systemic forms of treatment were used most commonly. In Pso, SpA, and connective tissue disease nonsteroidal anti-inflammatory drugs (NSAID) were the most frequently used treatment often in combination with other drugs.

**Conclusion:**

A considerable number of patients with IMIDs (16.9–27.5%) suffer from different diseases of the IMID group. They are frequently affected by accompanying illnesses and require interdisciplinary medical treatment.

Die Versorgung von Patienten mit chronischen Entzündungserkrankungen (engl. „immune-mediated inflammatory diseases“ [IMID]) ist herausfordernd und bezieht mehrere medizinische Fachgebiete mit ein. Auf Basis gesetzlicher Krankenkassendaten aus dem Kalenderjahr 2018 wurden gehäufte IMID-Kombinationen, das Auftreten weiterer Begleiterkrankungen sowie die spezifische Arzneimittelversorgung von IMID untersucht. Die Analysen deuten auf eine potenzielle Unterversorgung und eine teilweise nicht leitlinienkonforme Behandlung hin. Dies unterstreicht die Notwendigkeit eines umfassenden, interdisziplinären Managements von Patienten mit IMID.

## Hintergrund und Fragestellung

Chronische Entzündungserkrankungen bilden eine klinisch heterogene Erkrankungsgruppe, die immunologische Gemeinsamkeiten aufweisen können. Dazu zählen u. a. die Erkrankungen Psoriasis (Pso), Psoriasisarthritis (PsA), Spondylitis ankylosans (SpA), rheumatoide Arthritis (RA), Morbus Crohn (MC), Colitis ulcerosa (CU) und Kollagenosen.

Die Heterogenität der IMID stellt das Versorgungsmanagement, in welches diverse medizinische Fachdisziplinen involviert sind, vor große Herausforderungen. Insbesondere IMID-Kombinationen erfordern ein gutes interdisziplinäres, diagnostisches und therapeutisches Management und eine sehr gute Zusammenarbeit der verschiedenen Fachgruppen [20]. Laut Studien weisen Patienten mit IMID ein erhöhtes Risiko für weitere IMIDs auf und sind häufiger von Begleiterkrankungen (v. a. kardiovaskuläre Erkrankungen, Depressionen) betroffen [3, 20, 25, 30]. Je nach Erkrankungsschweregrad und zusätzlich auftretenden Begleiterkrankungen leiden Patienten mit IMID unter erheblichen Einschränkungen der Lebensqualität [14, 31]. Es ist erstrebenswert, dass Patienten mit IMID frühestmöglich von einem Spezialisten behandelt werden [14, 27, 32]. Arzneimitteltherapien sind für eine optimale Behandlung von wesentlicher Bedeutung und können das Fortschreiten von IMIDs aufhalten, Komplikationen der IMIDs, aber auch Komorbiditäten, wie z. B. kardiovaskuläre Ereignisse, verhindern, eine klinische Remission induzieren und die Lebensqualität verbessern [20, 27, 35].

### Ziel der Arbeit

Ziel der Studie war es, basierend auf Abrechnungsdaten der gesetzlichen Krankenversicherungen (GKV), die Krankheitslast und die aktuelle medikamentöse Versorgungsrealität bei Patienten mit IMIDs zu beschreiben. Die Krankheitslast wurde anhand von häufig auftretenden IMID-Kombinationen sowie anhand der Prävalenz weiterer Begleiterkrankungen im Vergleich zu einer Referenzpopulation beschrieben. Die Arzneimitteltherapie von IMID-Patienten wurde anhand der Verschreibung vordefinierter Gruppen medikamentöser Therapien beschrieben.

## Studiendesign und Untersuchungsmethoden

### Datenbasis und Studiendesign

Die Analyse basierte auf anonymisierten, alters- und geschlechtsadjustierten GKV-Abrechnungsdaten. Die daraus entnommene repräsentative Stichprobe hatte ein Volumen von 3.988.695 Versicherten, die der Datenbank des Instituts für angewandte Gesundheitsforschung Berlin (InGef) entstammte [4]. Zur Analyse der Krankheitslast und des Arzneimitteleinsatzes wurde eine retrospektive Querschnittstudie im Jahr 2018 durchgeführt.

### Identifikation der Studienpopulation

Durchgängig über den gesamten Beobachtungszeitraum bzw. bis zum Tod im Beobachtungszeitraum Versicherte mit mindestens 2 gesicherten ambulanten IMID-Diagnosen gleichzeitig in mindestens 2 Quartalen oder mindestens einer stationären Haupt- oder Nebendiagnose wurden als prävalente Patienten identifiziert. Die folgenden 7 Entitäten (gemäß ICD-10-GM) wurden separat und als aggregierte Gesamtgruppe berücksichtigt: Pso (L40.- ohne L40.5), PsA (L40.5 oder M07.-), RA (M05.- oder M06.-), SpA (M45.- und M46.-), CU (K51.-), MC (K50.-) und Kollagenosen. Letztere umfasste 4 Subgruppen: systemischer Lupus erythematodes (M32.-), systemische Sklerose (M34.-), Dermatomyositis/Polymyositis (M33.-) und andere Erkrankungen mit systemischer Beteiligung des Bindegewebes (z. B. M35.- ohne M35.3).

### IMID-Mehrfachdiagnosen und dokumentierte Diagnosen und Maßnahmen

In der Studienpopulation wurden IMID-Mehrfachdiagnosen innerhalb eines Jahres ermittelt und nach auftretender Häufigkeit eingestuft. Zudem wurden weitere nicht-IMID-bezogene Diagnosen und Maßnahmen anhand dokumentierter ICD-10-Codes ermittelt und einer korrespondierenden Referenzpopulation gegenübergestellt. Hierzu wurde je Entität eine alters- und geschlechtsbereinigte Referenzpopulation gebildet, die nicht von der jeweiligen Entität betroffen war.

### Therapieformen

Zur Darstellung der medikamentösen Versorgungsrealität wurden 8 verschiedene Therapieformen definiert und separat sowie als aggregierte Gesamtgruppe berücksichtigt (Tab. 1 und Tab. S1). Der Arzneimitteleinsatz wurde pro Entität jeweils anhand der Alters- und Geschlechtsverteilung beschrieben.

**Table 1:** 

Therapieform	Erläuterung
1. Biologika/JAKi	Kein Ausschluss weiterer Therapien
2. csDMARDs und klassische systemische Therapien	**Ausschluss von Biologika/JAKi**
	Kein Ausschluss weiterer definierter Therapien
3. Systemische Kortikosteroide als *Monotherapie*	**Ausschluss von Biologika/JAKi und DMARDs**
	Kein Ausschluss weiterer definierter Therapien
4. Systemische Kortikosteroide als *Kombinationstherapie*	**Kombination: Biologika/JAKi und/oder DMARDs**
	Kein Ausschluss weiterer definierter Therapien
5. NSAR – nichtsteroidale Antirheumatika	Ausschluss der Gruppen 1 bis 4 und 6
6. Coxibe (COX-2-Hemmer)	Ausschluss der Gruppen 1 bis 5
7. Nichtsteroidale Antipsoriatika zur dermalen Anwendung als *Monotherapie*	**Ausschluss von Biologika/JAKi und DMARDs**
	Kein Ausschluss weiterer definierter Therapien
8. Nichtsteroidale Antipsoriatika zur dermalen Anwendung als *Kombinationstherapie*	**Kombination: Biologika/JAKi und/oder DMARDs**
	Kein Ausschluss weiterer definierter Therapien
9. Gesamt	Disjunktive Angabe

## Ergebnisse

**Figure 1:**
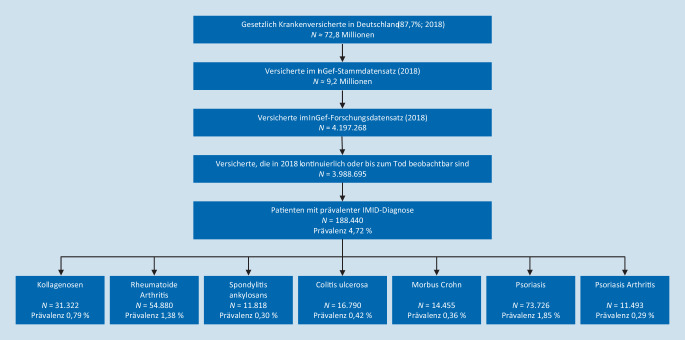

Im 1‑jährigen Beobachtungszeitraum wurden von insgesamt 3.988.695 GKV-Versicherten 188.440 Patienten mit mindestens einer IMID-Diagnose identifiziert (Prävalenz 4,7 pro 100.000 Personen, 95 %-Konfidenzintervall (CI): 4,704–4,745) (Abb. 1). Die folgenden Prävalenzen ergaben sich im Jahr 2018: 1,8 % für Pso, 1,3 % für RA, 0,4 % für CU, 0,4 % für MC, 0,3 % für SpA, 0,3 % für PsA und 0,8 % für Kollagenosen.

### IMID-Mehrfachdiagnosen und Begleiterkrankungen

**Figure 2:**
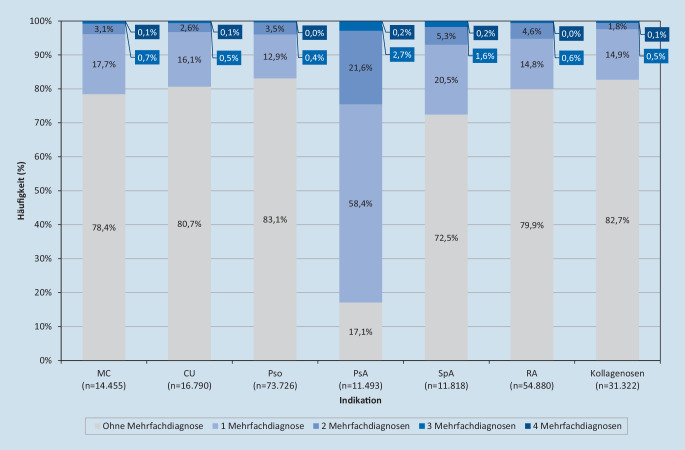

Es wiesen 17,1 % der PsA-Patienten keine, 58,4 % eine und 21,6 % zwei weitere IMID-Diagnosen auf (Abb. 2). In den weiteren Entitäten lag der Anteil der Patienten, die keine weitere dokumentierte IMID aufwiesen, zwischen 72,5 % (SpA) und 83,1 % (Pso). Zwischen 12,9 % (Pso) und 20,5 % (SpA) wiesen eine zusätzliche IMID-Diagnose auf.

**Figure 3:**
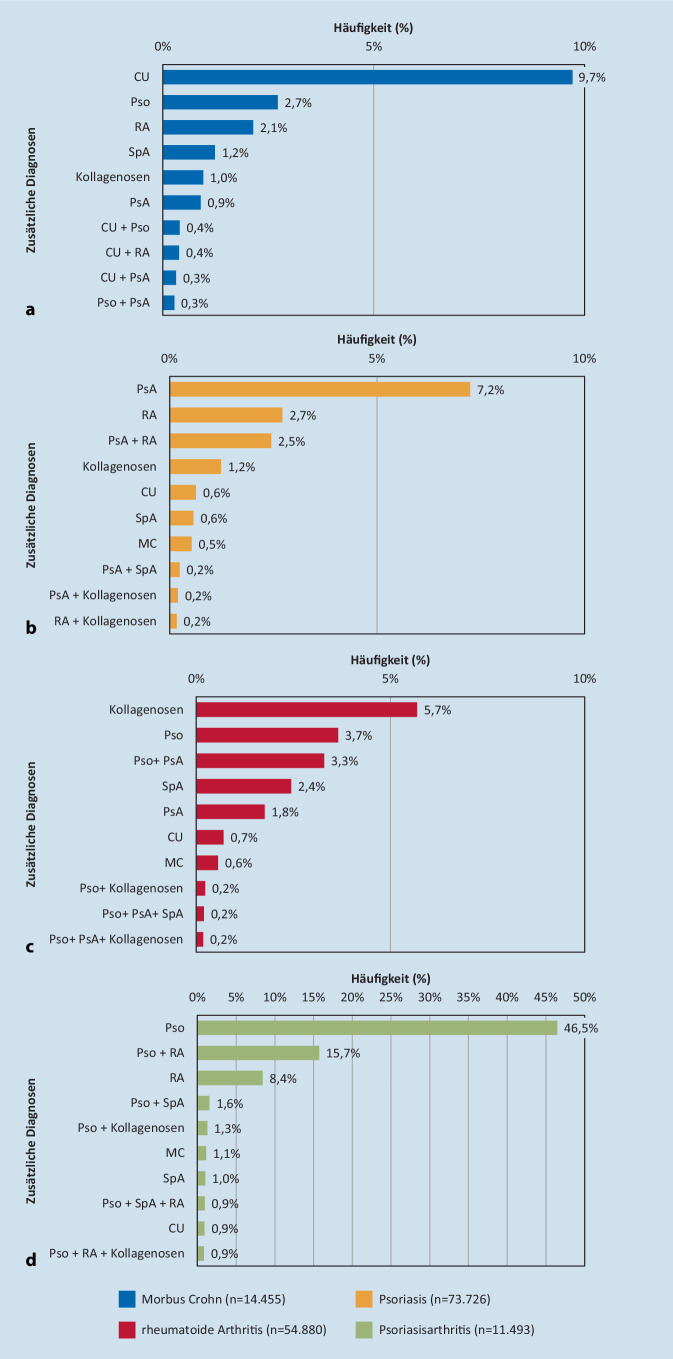

Bei MC-Patienten wurde im Beobachtungszeitraum am häufigsten eine CU dokumentiert (9,7 %), gefolgt von Pso (2,7 %) und RA (2,1 %) (Abb. 3). Bei 7,2 % der Pso-Patienten wurde ebenfalls eine PsA, bei 2,7 % eine RA und bei 2,5 % sowohl eine PsA als auch eine RA dokumentiert. Bei Patienten mit RA traten am häufigsten Kombinationen mit Kollagenosen (5,7 %), Pso (3,7 %) sowie Pso und PsA (3,3 %) auf. Bei 46,5 % der PsA-Patienten wurde zusätzlich eine Pso dokumentiert, gefolgt von der Kombination aus Pso und RA (15,7 %) und der RA (8,4 %).

Begleiterkrankungen traten bei Patienten mit IMID meist häufiger als in den jeweiligen Referenzpopulationen auf (Tab. S2). Rückenschmerzen und Hypertonie zählten innerhalb der Entitäten und der jeweiligen Referenzpopulationen zu den 10 häufigsten dokumentierten Diagnosen, wobei die beobachtete Häufigkeit dieser Diagnosen in jeder Entität höher als in der jeweiligen Referenzpopulation war (Tab. 2 und Tab. S2). Einige der in den Entitäten dominierenden Begleiterkrankungen (z. B. die depressive Episode bei Patienten mit MC, CU und SpA) zählten nicht zu den 10 häufigsten dokumentierten Diagnosen der entsprechenden Referenzpopulation. Bauch- und Beckenschmerzen fanden sich nur bei Patienten mit MC unter den Top-10-Diagnosen.

**Table 2:** 

Diagnose (ICD-Code)	Kohorte			Referenzpopulation		
	Rang	*n*	%	Rang	*n*	%
*Morbus Crohn (n* *=* *14.455)*						
Rückenschmerzen (M54)	1	5345	37,0	3	4377	30,3
Essenzielle (primäre) Hypertonie (I10)	2	5293	36,6	1	4692	32,5
Akkommodationsstörungen und Refraktionsfehler (H52)	4	3763	26,0	6	2849	19,7
Bauch- und Beckenschmerzen (R10)	6	3394	23,5	n/a	^a^	< 14,0
Depressive Episode (F32)	8	3091	21,4	n/a	^a^	< 14,0
*Colitis ulcerosa (n* *=* *16.790)*						
Essenzielle (primäre) Hypertonie (I10)	1	7213	43,0	1	6817	40,6
Rückenschmerzen (M54)	2	6331	37,7	2	5198	31,0
Störungen des Lipoproteinstoffwechsels und sonstige Lipidämien (E78)	4	5243	31,2	3	4882	29,1
Depressive Episode (F32)	8	3416	20,3	n/a	^a^	< 14,0
Somatoforme Störungen (F45)	10	3231	19,2	n/a	^a^	< 14,0
*Psoriasis (n* *=* *73.726)*						
Essenzielle (primäre) Hypertonie (I10)	1	41.871	56,8	1	34.864	47,3
Rückenschmerzen (M54)	2	30.792	41,8	2	24.017	32,6
Störungen des Lipoproteinstoffwechsels und sonstige Lipidämien (E78)	3	30.724	41,7	3	25.275	34,3
Spondylose (M47)	10	14.982	20,3	n/a	^a^	< 15,0
*Psoriasisarthritis (n* *=* *11.493)*						
Psoriasis (L40)	1	8447	73,5	n/a	^a^	< 15,0
Essenzielle (primäre) Hypertonie (I10)	2	6445	56,1	2	5106	44,4
Rückenschmerzen (M54)	3	5643	49,1	3	3776	32,9
Sonstige Krankheiten des Weichteilgewebes, anderenorts nicht klassifiziert (M79)	6	4061	35,3	n/a	^a^	< 15,0
Sonstige chronische Polyarthritis (M06)	7	3855	33,5	n/a	^a^	< 15,0
Spondylose (M47)	9	3103	27,0	n/a	^a^	< 15,0
*Spondylitis ankylosans (n* *=* *11.818)*						
Essenzielle (primäre) Hypertonie (I10)	1	6227	52,7	1	5259	44,5
Rückenschmerzen (M54)	2	5983	50,6	2	3801	32,2
Störungen des Lipoproteinstoffwechsels und sonstige Lipidämien (E78)	3	4408	37,3	3	3857	32,6
Spondylose (M47)	6	3370	28,5	n/a	^a^	< 14,0
Sonstige Krankheiten des Weichteilgewebes, anderenorts nicht klassifiziert (M79)	7	3253	27,5	n/a	^a^	< 14,0
Schmerz, anderenorts nicht klassifiziert (R52)	8	3016	25,5	n/a	^a^	< 14,0
Depressive Episode (F32)	10	2781	23,5	n/a	^a^	< 14,0
*Rheumatoide Arthritis (n* *=* *54.880)*						
Essenzielle (primäre) Hypertonie (I10)	1	35.669	65,0	1	31.070	56,6
Rückenschmerzen (M54)	2	27.915	50,9	2	19.338	35,2
Störungen des Lipoproteinstoffwechsels und sonstige Lipidämien (E78)	3	25.466	46,4	3	22.167	40,4
Sonstige Krankheiten des Weichteilgewebes, anderenorts nicht klassifiziert (M79)	4	22.321	40,7	n/a	^a^	< 17,0
Spondylose (M47)	8	16.481	30,0	n/a	^a^	< 17,0
Gonarthrose (Arthrose des Kniegelenkes) (M17)	9	16.078	29,3	n/a	^a^	< 17,0
Schmerz, anderenorts nicht klassifiziert (R52)	10	16.022	29,2	n/a	^a^	< 17,0

^a^ Diagnosen nicht im Top-10-Ranking der Referenzpopulation enthalten

### Therapieformen

**Figure 4:**
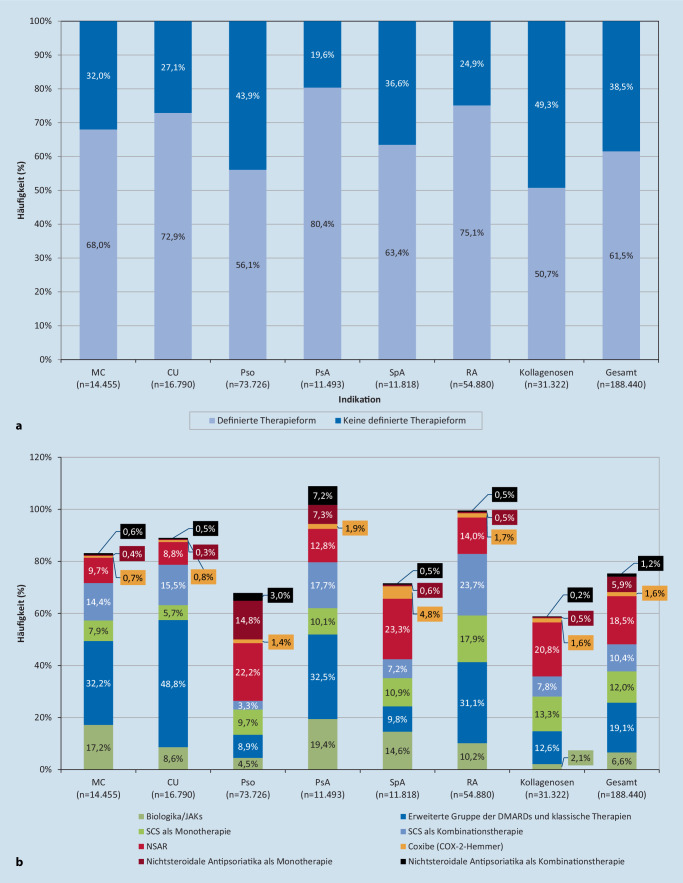

Insgesamt erhielten 38,5 % der Patienten mit IMID keine der definierten medikamentösen Therapieformen. In den Entitäten lag der Anteil zwischen 19,6 % (PsA) und 49,3 % (Kollagenosen) (Abb. 4a). Am häufigsten eingesetzte Therapieformen in der Gesamtpopulation aller IMIDs waren die erweiterte Gruppe der csDMARDs und klassischen systemischen Therapien (19,1 %), gefolgt von nichtsteroidalen Antirheumatika (NSAR) (18,5 %) und systemischen Kortikosteroiden (SCS), entweder als Monotherapie (12,0 %) oder in Kombination mit Biologika/Januskinaseinhibitoren (JAKi) (10,4 %) (Abb. 4b).

Bei Patienten mit MC, CU, RA und PsA, bei denen mindestens eine Abgabe der definierten Therapieformen dokumentiert wurde, war die häufigste Therapieform die erweiterte Gruppe der csDMARDs und klassischen systemischen Therapien mit einem Anteil von jeweils über 30 % (Abb. 4b). Bei Patienten mit Pso, SpA und Kollagenosen waren NSAR mit einem Anteil von über 20 % die häufigste Therapieform. Nichtsteroidale Antipsoriatika zur dermalen Anwendung wurden am häufigsten bei Patienten mit Pso (14,8 %) und PsA (7,3 %) eingesetzt. Biologika/JAKi wurden zu Anteilen von 19,4 % PsA, 17,2 % CED, 14,6 % SpA, 10,2 % RA, 8,6 % CU, 4,5 % Pso und 2,1 % Kollagenosen verordnet. Zusätzlich zur Variabilität im Hinblick auf den Einsatz der verschiedenen Therapieformen wurden teilweise auch in den einzelnen Entitäten Alters- und Geschlechtsunterschiede beobachtet (Tab. S3–S10).

## Diskussion

Die Analyse widmete sich den Fragen, welche IMID-Kombinationen gehäuft auftreten, in welchem Ausmaß Patienten mit IMID von weiteren Begleiterkrankungen betroffen sind und inwieweit spezifische medikamentöse Therapieformen in dieser Patientenpopulation eingesetzt werden.

Die für die einzelnen Erkrankungen berechneten Prävalenzen lagen im Bereich zuvor publizierter Daten mit 1,8 % für Pso (2,0–2,1 % [18]), 0,3 % für PsA (0,2–0,3 % [7, 33]), 0,3 % für SpA (0,1–0,8 [7, 33]), 1,3 % für RA (0,8–1,2 % [2]), 0,8 % für Kollagenosen (0,2–0,3 % [38]), 0,4 % für CU (0,5 % [29]) und 0,4 % für MC (0,32 % [29]).

Die häufigsten in Kombination mit PsA auftretenden IMID waren Pso und RA. Eine hohe Kombinationsrate von PsA und Pso ist erwartbar, da die Pso häufig einer PsA vorausgeht oder gleichzeitig auftritt [3]. Die in der Literatur berichtete Prävalenz von PsA bei Pso-Patienten schwankt stark zwischen 6 und 42 % und liegt damit teilweise deutlich über der Prävalenz in dieser Studie [11, 15]. Es ist daher möglich, dass Patienten mit Pso bezüglich PsA teilweise unzureichend diagnostiziert und therapiert werden. Das beobachtete gemeinsame Auftreten von PsA und RA lässt hingegen eher Fehldiagnosen oder Schwierigkeiten in der klinischen Differenzierung vermuten. In seltenen Fällen, wenn z. B. eine Pso erst nach Beginn der Arthritis auftritt, kann eine Unterscheidung von anderen Gelenkerkrankungen (z. B. RA) erschwert sein [19]. Zudem sind einzelne Fälle von Overlap-Syndromen mit der entsprechenden HLA-Konstellation beschrieben.

Auffallend war, dass fast 10 % der Patienten mit MC auch eine CU-Diagnose aufwiesen. Das gemeinsame Auftreten von MC- und CU-Diagnosen könnte auf eine erschwerte diagnostische Unterscheidung aufgrund ähnlicher Symptome und Krankheitsverläufe zurückzuführen sein [37]. Es besteht die Notwendigkeit, basierend auf Veränderungen im endoskopischen Bild, die Diagnose zu revidieren.

Patienten mit Pso, PsA, SpA und RA wiesen oft Begleiterkrankungen wie Hypertonie auf. Auch in der Literatur wurde von einem erhöhten Hypertonierisiko und einem erhöhten kardiovaskulären Risiko bei Patienten mit Pso sowie RA, SpA und PsA berichtet [1, 8, 10, 30]. Laut der European Alliance of Associations for Rheumatology (EULAR) sollen Patienten mit RA und anderen entzündlichen Gelenkerkrankungen wie SpA und PsA deshalb besonders auf dieses Risiko hin beobachtet und ggf. therapiert werden [1]. Weiterhin litten Patienten mit MC, CU und SpA im Vergleich zu den jeweiligen Referenzpopulationen häufiger an einer depressiven Episode. Dies kann in Übereinstimmung mit aktuellen Studien auf erhöhte psychische Belastungen mit chronischem Stress, veränderter Stressbewältigung/Stressreaktion, Einnahme oder Absetzen von Medikamenten (z. B. Glukokortikoide) oder Veränderung von Zytokinen im Rahmen der Grunderkrankung (welche auch in der Pathogenese von Depressionen eine wichtige Rolle spielen) hindeuten [6, 17, 21, 26].

DMARDs werden bei der Behandlung von PsA und RA als Erstlinientherapie empfohlen [13, 16, 34]. Eine Behandlung mit Biologika oder JAKi wird in der Regel bei Patienten mit einem schweren Krankheitsverlauf und bei Patienten, die auf die erweiterte Gruppe der csDMARDs und klassische systemische Therapien (Tab. S1) unzureichend angesprochen haben, empfohlen [16, 34]. Zwar waren DMARDs und klassische systemische Therapien sowohl bei RA- als auch bei PsA-Patienten die häufigste verordnete Therapieform, dennoch fiel der Anteil dieser Patienten mit entsprechender Verordnung eher gering aus. Auch erhielten in dieser Studie nur etwa 10 % der RA-Patienten eine Verordnung mit Biologika/JAKi, während z. B. in Irland und den Niederlanden bereits im Jahr 2010 etwa 35 % der RA-Patienten mit Biologika therapiert wurden [23].

NSAR waren bei Patienten mit SpA die häufigste angewandte Therapieform. Dies deckt sich mit der S3-Leitlinie, in der NSAR als Erstlinientherapie empfohlen werden [22]. Biologika werden hingegen bei Patienten mit schwerem Krankheitsverlauf und unzureichendem Ansprechen auf eine NSAR-Therapie empfohlen [22]. Der Anteil der mit Biologika/JAKi behandelten Patienten fiel geringer aus. NSAR werden in den kürzlich publizierten GRAPPA-Empfehlungen bei PsA nur noch bedingt für die Arthritismanifestation empfohlen, können jedoch bei Enthesitiden zur Anwendung kommen [9].

Der Anteil der IMID-Patienten mit Verordnungen von Biologika/JAKi fiel insgesamt gering aus. Zum einen waren JAKi 2018 noch nicht in allen betrachteten Entitäten zugelassen [5, 36]. Zum anderen werden Biologika nur bei schweren Krankheitsverläufen sowie bei unzureichendem Ansprechen auf andere konventionelle Therapieformen empfohlen. Da die Information zum Krankheitsschweregrad in dieser Analyse nicht vorlag, ist eine Einordnung der beobachteten Anteile nur begrenzt möglich.

Seit 2004 wurden diverse Biologika für die Pso-Therapie zugelassen, deren Einsatz neben csDMARDs und klassischen systemischen Therapien zur Behandlung einer mittelschweren und schweren Psoriasis vulgaris empfohlen wird [24, 28]. Dennoch waren NSAR bei Pso-Patienten die häufigste verordnete Therapieform. Auch bei MC und CU fiel auf, dass ein wesentlicher Anteil der Patienten mit NSAR behandelt wurde, obwohl diese aufgrund ihrer schubauslösenden Wirkung bei chronisch entzündlichen Darmerkrankungen als kontraindiziert gelten [12]. Da NSAR auch rezeptfrei verfügbar sind, erscheint dieser Anteil an NSAR-Verordnungen alarmierend. Eine Erklärung könnte die Behandlung von Gelenkschmerzen sein, z. B. bei einer zeitgleich vorliegenden RA, ohne Berücksichtigung des Gesamtzustandes des Patienten. Dies verdeutlicht die Notwendigkeit eines interdisziplinären Therapiemanagements bei Patienten mit IMID.

Die Stärke der Studie stellt die zugrunde liegende umfangreiche repräsentative Datenbasis dar, welche den Vorzug bietet, das Versorgungsgeschehen authentisch abzubilden. Aufgrund des Querschnittdesigns sind Analysen zum Krankheits- und Therapieverlauf und eine kausale Interpretation der Ergebnisse nicht zulässig. Gleichzeitig stellt die Art des Datensatzes auch dessen größte Einschränkung dar. Bei der Erfassung lässt sich naturgemäß nicht nachvollziehen, welche Facharztgruppe die Diagnose kodiert hat. Der teilweise hohe Anteil an Patienten, die offenbar nicht leitliniengerecht behandelt werden, könnte ein Hinweis darauf sein, dass die Diagnose in vielen Fällen nicht durch die entsprechende Facharztgruppe gestellt bzw. kodiert wurde.

## Schlussfolgerung

Die Studie liefert aktuelle Real-World-Evidence zur Krankheitslast und medikamentösen Versorgungssituation von IMID-Patienten basierend auf einer hinsichtlich Alter und Geschlecht repräsentativen GKV-Versichertenstichprobe. IMID-Patienten leiden teilweise an mehreren IMID und häufiger an Begleiterkrankungen. Die Ergebnisse deuten auf eine potenzielle Unterversorgung und eine teilweise nicht leitlinienkonforme Behandlung hin und betonen die Notwendigkeit einer besseren, ganzheitlichen, interdisziplinären Behandlung von Patienten mit IMID. Eine optimale Therapiesteuerung ist bei IMID von zentraler Bedeutung, um irreversible Organschädigungen zu vermeiden. Bei der Arzneimitteltherapie sollten daher neben dem Schweregrad und Therapieverlauf auch auftretende IMID-Kombinationen und Begleiterkrankungen berücksichtigt werden. Zukünftig werden Längsschnittstudien benötigt, um Einblicke in den Krankheits- und Therapieverlauf bei IMID-Patienten zu gewinnen. Zu erwähnen ist noch, dass seit 2018, dem Jahr der Studienpopulationserfassung, eine Reihe neuer Biologika und JAKi für IMIDs zugelassen wurde, sodass sich die Versorgungssituation 2022 unterscheiden könnte.

## Fazit für die Praxis

- Die Prävalenz der betrachteten IMID lag 2018 bei 4,7 %.
- Patienten mit IMID litten teilweise an mehreren IMID und wiesen häufig Begleiterkrankungen wie kardiovaskuläre Risikoerkrankungen und Depressionen auf.
- Betroffene wurden teilweise offenbar nicht leitlinienkonform behandelt.
- Im internationalen Vergleich wurden Biologika/JAKi in Deutschland bei IMID eher selten angewendet, was auf eine Unterversorgung bei Betroffenen hindeutet.
- Die Ergebnisse betonen die Notwendigkeit einer besseren, ganzheitlichen, interdisziplinären Behandlung von Patienten mit IMID.

### Supplementary Information




