# Supplementary material for: Immune-mediated inflammatory diseases in Germany: A cross-sectional analysis of comorbidities and pharmacotherapy
Source: Z Rheumatol. 2023 Jan 4;83(3):200–9. [Article in German] doi: 10.1007/s00393-022-01306-1 (PMC10972917; doi:10.1007/s00393-022-01306-1)
Supplement: Supplementary file 1 [file 393_2022_1306_MOESM1_ESM.pdf]

Zusatzmaterial

Chronische Entzündungserkrankungen in Deutschland

Eine Querschnittsanalyse über Begleiterkrankungen und Arzneimitteleinsatz

Jan Leipe, Renate Schmelz, Gabriela Riemekasten, Diamant Thaçi, Jörg Henes, Knut Schäkel, Andreas Pinter, Michael Sticherling, Joanna Wegner, Stefano Fusco, Miriam Linke, Valeria Weber, Karina C. Manz, Holger Bartz, Marit Roecken, Sandra Schmidt, Bimba F. Hoyer

Tab. S1: Definierte Therapieformen und dazugehörige Wirkstoffe

Gruppe	Klassifizierung	ATC Code	Wirkstoff
Biologika/JAKi	Selektive Immunsuppressiva	L04AA24	Abatacept
		L04AA26	Belimumab
		L04AA33	Vedolizumab
	TNF-alpha-Inhibitor	L04AB01	Etanercept
		L04AB02	Infliximab
		L04AB04	Adalimumab
		L04AB05	Certolizumab pegol
		L04AB06	Golimumab
	Interleukin-Inhibitor	L04AC03	Anakinra
		L04AC05	Ustekinumab
		L04AC07	Tocilizumab
		L04AC10	Secukinumab
		L04AC12	Brodalumab
		L04AC13	Ixekizumab
		L04AC14	Sarilumab
		L04AC16	Guselkumab
		L04AC17	Tildrakizumab
		L04AC18	Risankizumab
	Monoklonale Antikörper	L01XC02	Rituximab
	JAK-Inhibitor	L04AA37	Baricitinib
		L04AA29	Tofacitinib
csDMARDs & klassische systemische Therapien	Intestinale Antiphlogistika	A07EC01	Sulfasalazin
		A07EC02	Mesalazin
	Antipsoriatika zur systemischen Anwendung	D05BB02	Acitretin
		D05BX02	Dimethylfumarat
		D05BX51	(Dimethylphomarat) Fumarsäure-Derivate, Kombinationen
	Immunsuppressiva	L04AA32	Apremilast
		L04AA13	Leflunomid
		L04AD01	Cyclosporin A
		L04AX01	Azathioprin

Gruppe	Klassifizierung	ATC Code	Wirkstoff
		L04AX03	Methotrexat
	Antimetaboliten	L01BA01	Methotrexat
	Spezifische Antirheumatika	M01CX01	Methotrexat
		M01CX02	Sulfasalazin
	Malariamittel	P01BA02	Hydroxychloroquin(sulfat)
Systemische Corticosteroide	Systemische Corticosteroide (als Monotherapie oder als Kombination)	H02AB01	Betamethason
		H02AB02	Dexamethason
		H02AB03	Fluocortolon
		H02AB04	Methylprednisolon
		H02AB05	Paramethason
		H02AB06	Prednisolon
		H02AB07	Prednison
		H02AB08	Triamcinolon
		H02AB09	Hydrocortison
		H02AB10	Cortison
		H02AB11	Prednyliden
		H02AB12	Rimexolon
		H02AB13	Deflazacort
		H02AB14	Cloprednol
		H02AB15	Meprednison
		H02AB17	Cortivazol
		H02AB51	Betamethason-Depot
		H02AB54	Methylprednisolon-Depot
		H02AB56	Prednisolon-Depot
		H02AB58	Triamcinolon-Depot
		H02BX01	Methylprednisolon, Kombinationen
		H02BX02	Dexamethason, Kombinationen
		H02BX06	Prednisolon, Kombinationen
		H02BX08	Triamcinolon, Kombinationen
		H02BX09	Betamethason, Kombinationen
Nicht-steroidale Antirheumatika (NSAR)	Essigsäure-Derivate und verwandte Substanzen	M01AB01	Indometacin
		M01AB02	Sulindac
		M01AB03	Tolmetin
		M01AB04	Zomepirac
		M01AB05	Diclofenac
		M01AB06	Alclofenac
		M01AB07	Bumadizon
		M01AB08	Etodolac
		M01AB09	Lonazolac
		M01AB10	Fentiazac
		M01AB11	Acemetacin
		M01AB12	Difenpiramid
		M01AB13	Oxametacin
		M01AB14	Proglumetacin
		M01AB15	Ketorolac
		M01AB16	Aceclofenac

Gruppe	Klassifizierung	ATC Code	Wirkstoff
		M01AB17	Bufexamac
		M01AB19	Carbamoylphenoxyessigsäure
		M01AB51	Indometacin, Kombinationen
		M01AB55	Diclofenac, Kombinationen
		M01AB69	Carbamoylphenoxyessigsäure, Kombinationen
	Propionsäure-Derivate	M01AE01	Ibuprofen
		M01AE02	Naproxen
		M01AE03	Ketoprofen
		M01AE04	Fenoprofen
		M01AE05	Fenbufen
		M01AE06	Benoxaprofen
		M01AE07	Suprofen
		M01AE08	Pirprofen
		M01AE09	Flurbiprofen
		M01AE10	Indoprofen
		M01AE11	Tiaprofensäure
		M01AE12	Oxaprozin
		M01AE13	Ibuproxam
		M01AE14	Dexibuprofen
		M01AE15	Flunoxaprofen
		M01AE16	Alminoprofen
		M01AE17	Dexketoprofen
		M01AE18	Naproxcinod
		M01AE20	Carprofen
		M01AE51	Ibuprofen, Kombinationen
		M01AE52	Naproxen und Esomeprazol
		M01AE53	Ketoprofen, Kombinationen
		M01AE56	Naproxen und Misoprostol
Coxibe	Coxibe	M01AH01	Celecoxib
		M01AH02	Rofecoxib
		M01AH03	Valdecoxib
		M01AH04	Parecoxib
		M01AH05	Etoricoxib
		M01AH06	Lumiracoxib
Nicht-steroidale Antipsoriatika zur dermalen Anwendung	Antipsoriatika zur dermalen Anwendung (als Monotherapie oder als Kombination)	D05AC01	Dithranol
		D05AC51	Dithranol, Kombinationen
		D05AX02	Calcipotriol
		D05AX03	Calcitriol
		D05AX04	Tacalcitol
		D05AX05	Tazaroten
		D05AX22	Calcipotriol und Betamethason
		D05AX52	Calcipotriol, Kombinationen
		D05AX56	Salicylsäure, Kombinationen

Tab. S2: Top 10-Ranking der dokumentierten Diagnosen/Maßnahmen pro Indikation und der korrespondierenden Referenzpopulation

Indikation	Kohorte				Referenzpopulation			
	ICD-Code	Diagnose/Maßnahme	n	%	ICD-Code	Diagnose/Maßnahme	n	%
MC (n=14.455)	M54	Rückenschmerzen	5.345	37,0	I10	Essentielle (primäre) Hypertonie	4.692	32,46
	I10	Essentielle (primäre) Hypertonie	5.293	36,6	Z12	Spezielle Verfahren zur Untersuchung auf Neubildungen	4.414	30,54
	Z12	Spezielle Verfahren zur Untersuchung auf Neubildungen	4.924	34,1	M54	Rückenschmerzen	4.377	30,28
	H52	Akkommodationsstörungen und Refraktionsfehler	3.763	26,0	E78	Störungen des Lipoproteinstoffwechsels und sonstige Lipidämien	3.467	23,98
	J06	Akute Infektionen an mehreren oder nicht näher bezeichneten Lokalisationen der oberen Atemwege	3.585	24,8	J06	Akute Infektionen an mehreren oder nicht näher bezeichneten Lokalisationen der oberen Atemwege	3.034	20,99
	R10	Bauch- und Beckenschmerzen	3.394	23,5	H52	Akkommodationsstörungen und Refraktionsfehler	2.849	19,71
	E78	Störungen des Lipoproteinstoffwechsels und sonstige Lipidämien	3.296	22,8	Z30	Kontrazeptive Maßnahmen	2.676	18,51
	F32	Depressive Episode	3.091	21,4	N89	Sonstige nichtentzündliche Krankheiten der Vagina	2.546	17,61
	Z25	Notwendigkeit der Impfung [Immunisierung] gegen andere einzelne Viruskrankheiten	2.855	19,8	Z00	Allgemeinuntersuchung und Abklärung bei Personen ohne Beschwerden oder angegebene Diagnose	2.313	16,00
	Z30	Kontrazeptive Maßnahmen	2.848	19,7	Z01	Sonstige spezielle Untersuchungen und Abklärungen bei Personen ohne Beschwerden oder angegebene Diagnose	2.076	14,36
CU (n=16.790)	I10	Essentielle (primäre) Hypertonie	7.213	43,0	I10	Essentielle (primäre) Hypertonie	6.817	40,60
	M54	Rückenschmerzen	6.331	37,7	M54	Rückenschmerzen	5.198	30,96
	Z12	Spezielle Verfahren zur Untersuchung auf Neubildungen	5.646	33,6	E78	Störungen des Lipoproteinstoffwechsels und sonstige Lipidämien	4.882	29,08
	E78	Störungen des Lipoproteinstoffwechsels und sonstige Lipidämien	5.243	31,2	Z12	Spezielle Verfahren zur Untersuchung auf Neubildungen	4.720	28,11
	H52	Akkommodationsstörungen und Refraktionsfehler	4.881	29,1	H52	Akkommodationsstörungen und Refraktionsfehler	3.844	22,89
	Z25	Notwendigkeit der Impfung [Immunisierung] gegen andere einzelne Viruskrankheiten	3.731	22,2	J06	Akute Infektionen an mehreren oder nicht näher bezeichneten Lokalisationen der oberen Atemwege	3.145	18,73

Indikation	Kohorte				Referenzpopulation			
	ICD-Code	Diagnose/Maßnahme	n	%	ICD-Code	Diagnose/Maßnahme	n	%
	J06	Akute Infektionen an mehreren oder nicht näher bezeichneten Lokalisationen der oberen Atemwege	3.723	22,2	Z00	Allgemeinuntersuchung und Abklärung bei Personen ohne Beschwerden oder angegebene Diagnose	2.852	16,99
	F32	Depressive Episode	3.416	20,3	Z25	Notwendigkeit der Impfung [Immunisierung] gegen andere einzelne Viruskrankheiten	2.540	15,13
	Z00	Allgemeinuntersuchung und Abklärung bei Personen ohne Beschwerden oder angegebene Diagnose	3300	19,65	Z30	Kontrazeptive Maßnahmen	2.296	13,67
	F45	Somatoforme Störungen	3.231	19,2	N89	Sonstige nichtentzündliche Krankheiten der Vagina	2.295	13,67
Pso (n=73.726)	I10	Essentielle (primäre) Hypertonie	41.871	56,8	I10	Essentielle (primäre) Hypertonie	34.864	47,29
	M54	Rückenschmerzen	30.792	41,8	E78	Störungen des Lipoproteinstoffwechsels und sonstige Lipidämien	25.275	34,28
	E78	Störungen des Lipoproteinstoffwechsels und sonstige Lipidämien	30.724	41,7	M54	Rückenschmerzen	24.017	32,58
	Z12	Spezielle Verfahren zur Untersuchung auf Neubildungen	25.495	34,6	Z12	Spezielle Verfahren zur Untersuchung auf Neubildungen	21.535	29,21
	H52	Akkommodationsstörungen und Refraktionsfehler	23.411	31,8	H52	Akkommodationsstörungen und Refraktionsfehler	18.595	25,22
	Z25	Notwendigkeit der Impfung [Immunisierung] gegen andere einzelne Viruskrankheiten	17.697	24	Z00	Allgemeinuntersuchung und Abklärung bei Personen ohne Beschwerden oder angegebene Diagnose	14.105	19,13
	E66	Adipositas	16.878	22,9	Z25	Notwendigkeit der Impfung [Immunisierung] gegen andere einzelne Viruskrankheiten	13.689	18,57
	Z00	Allgemeinuntersuchung und Abklärung bei Personen ohne Beschwerden oder angegebene Diagnose	16.232	22,0	J06	Akute Infektionen an mehreren oder nicht näher bezeichneten Lokalisationen der oberen Atemwege	12.365	16,77
	E11	Diabetes mellitus, Typ 2	15.508	21,0	E11	Diabetes mellitus, Typ 2	11.514	15,62
	M47	Spondylose	14.982	20,3	E66	Adipositas	10.917	14,81
PsA (n=11.493)	L40	Psoriasis	8.447	73,50	I10	Essentielle (primäre) Hypertonie	5.106	44,43
	I10	Essentielle (primäre) Hypertonie	6.445	56,08	E78	Störungen des Lipoproteinstoffwechsels und sonstige Lipidämien	3.792	32,99
	M54	Rückenschmerzen	5.643	49,10	M54	Rückenschmerzen	3.776	32,85
	E78	Störungen des Lipoproteinstoffwechsels und sonstige Lipidämien	4.545	39,55	Z12	Spezielle Verfahren zur Untersuchung auf Neubildungen	3.687	32,08

Indikation	Kohorte				Referenzpopulation			
	ICD-Code	Diagnose/Maßnahme	n	%	ICD-Code	Diagnose/Maßnahme	n	%
	Z12	Spezielle Verfahren zur Untersuchung auf Neubildungen	4.291	37,34	H52	Akkommodationsstörungen und Refraktionsfehler	2.751	23,94
	M79	Sonstige Krankheiten des Weichteilgewebes, anderenorts nicht klassifiziert	4.061	35,33	Z00	Allgemeinuntersuchung und Abklärung bei Personen ohne Beschwerden oder angegebene Diagnose	2.129	18,52
	M06	Sonstige chronische Polyarthritis	3.855	33,54	J06	Akute Infektionen an mehreren oder nicht näher bezeichneten Lokalisationen der oberen Atemwege	1.993	17,34
	H52	Akkommodationsstörungen und Refraktionsfehler	3.854	33,53	Z25	Notwendigkeit der Impfung [Immunisierung] gegen andere einzelne Viruskrankheiten	1.986	17,28
	M47	Spondylose	3.103	27,00	F32	Depressive Episode	1.703	14,82
	Z25	Notwendigkeit der Impfung [Immunisierung] gegen andere einzelne Viruskrankheiten	3.004	26,14	E66	Adipositas	1.695	14,75
SpA (n=11.818)	I10	Essentielle (primäre) Hypertonie	6.227	52,7	I10	Essentielle (primäre) Hypertonie	5.259	44,50
	M54	Rückenschmerzen	5.983	50,6	E78	Störungen des Lipoproteinstoffwechsels und sonstige Lipidämien	3.857	32,64
	E78	Störungen des Lipoproteinstoffwechsels und sonstige Lipidämien	4.408	37,3	M54	Rückenschmerzen	3.801	32,16
	Z12	Spezielle Verfahren zur Untersuchung auf Neubildungen	3.954	33,5	Z12	Spezielle Verfahren zur Untersuchung auf Neubildungen	3.346	28,31
	H52	Akkommodationsstörungen und Refraktionsfehler	3.938	33,3	H52	Akkommodationsstörungen und Refraktionsfehler	2.866	24,25
	M47	Spondylose	3.370	28,5	Z00	Allgemeinuntersuchung und Abklärung bei Personen ohne Beschwerden oder angegebene Diagnose	2.160	18,28
	M79	Sonstige Krankheiten des Weichteilgewebes, anderenorts nicht klassifiziert	3.253	27,5	J06	Akute Infektionen an mehreren oder nicht näher bezeichneten Lokalisationen der oberen Atemwege	2.017	17,07
	R52	Schmerz, anderenorts nicht klassifiziert	3.016	25,5	Z25	Notwendigkeit der Impfung [Immunisierung] gegen andere einzelne Viruskrankheiten	2.005	16,97
	Z25	Notwendigkeit der Impfung [Immunisierung] gegen andere einzelne Viruskrankheiten	2.859	24,2	E11	Diabetes mellitus, Typ 2	1.731	14,65
	F32	Depressive Episode	2.781	23,5	E66	Adipositas	1.656	14,01
RA (n=54.880)	I10	Essentielle (primäre) Hypertonie	35.669	65,0	I10	Essentielle (primäre) Hypertonie	31.070	56,61
	M54	Rückenschmerzen	27.915	50,9	E78	Störungen des Lipoproteinstoffwechsels und sonstige Lipidämien	22.167	40,39

Indikation	Kohorte				Referenzpopulation			
	ICD-Code	Diagnose/Maßnahme	n	%	ICD-Code	Diagnose/Maßnahme	n	%
	E78	Störungen des Lipoproteinstoffwechsels und sonstige Lipidämien	25.466	46,4	M54	Rückenschmerzen	19.338	35,24
	M79	Sonstige Krankheiten des Weichteilgewebes, anderenorts nicht klassifiziert	22.321	40,7	Z12	Spezielle Verfahren zur Untersuchung auf Neubildungen	17.366	31,64
	H52	Akkommodationsstörungen und Refraktionsfehler	22.143	40,3	H52	Akkommodationsstörungen und Refraktionsfehler	16.561	30,18
	Z12	Spezielle Verfahren zur Untersuchung auf Neubildungen	19.531	35,6	Z25	Notwendigkeit der Impfung [Immunisierung] gegen andere einzelne Viruskrankheiten	13.218	24,09
	Z25	Notwendigkeit der Impfung [Immunisierung] gegen andere einzelne Viruskrankheiten	16.985	31,0	Z00	Allgemeinuntersuchung und Abklärung bei Personen ohne Beschwerden oder angegebene Diagnose	11.223	20,45
	M47	Spondylose	16.481	30,0	E11	Diabetes mellitus, Typ 2	10.397	18,94
	M17	Gonarthrose [Arthrose des Kniegelenkes]	16.078	29,3	N95	Klimakterische Störungen	9.595	17,48
	R52	Schmerz, anderenorts nicht klassifiziert	16.022	29,2	E04	Sonstige nichttoxische Struma	9.476	17,27

Tab. S3: Demografie der Therapieformen – Total

Therapiegruppe	Altersgruppen	Weiblich		Männlich		Gesamt	
		n	%	n	%	n	%
Biologika/JAKi	1-10	16	0,2%	8	0,1%	24	0,2%
	11-20	203	3,0%	177	3,1%	380	3,0%
	21-30	613	9,1%	590	10,2%	1.203	9,6%
	31-40	853	12,7%	944	16,3%	1.797	14,4%
	41-50	1.011	15,0%	1.165	20,2%	2.176	17,4%
	51-60	1.660	**24,7%**	1.547	**26,8%**	3.207	**25,6%**
	61-70	1.416	21,0%	883	15,3%	2.299	18,4%
	71-80	788	11,7%	380	6,6%	1.168	9,3%
	81-90	165	2,5%	83	1,4%	248	2,0%
	≥ 91	<5	*	<5	*	<5	*
	Gesamt	6.727	100,0%	5.778	100,0%	12.505	100,0%
csDMARDs & klassische systemische Therapien	1-10	39	0,2%	30	0,2%	69	0,2%
	11-20	272	1,3%	245	1,7%	517	1,4%
	21-30	977	4,5%	792	5,5%	1.769	4,9%
	31-40	1.741	8,1%	1.353	9,4%	3.094	8,6%
	41-50	2.632	12,2%	1.988	13,8%	4.620	12,8%
	51-60	4.873	22,6%	3.537	**24,5%**	8.410	**23,3%**
	61-70	5.206	**24,1%**	3.190	22,1%	8.396	23,3%
	71-80	4.020	18,6%	2.435	16,8%	6.455	17,9%
	81-90	1.691	7,8%	852	5,9%	2.543	7,1%
	≥ 91	112	0,5%	35	0,2%	147	0,4%
	Gesamt	21.563	100,0%	14.457	100,0%	36.020	100,0%
Systemische Corticosteroide als Monotherapie	1-10	20	0,1%	25	0,3%	45	0,2%
	11-20	65	0,5%	31	0,4%	96	0,4%
	21-30	272	2,0%	184	2,1%	456	2,0%
	31-40	613	4,4%	335	3,8%	948	4,2%
	41-50	1.067	7,7%	739	8,5%	1.806	8,0%
	51-60	2.494	17,9%	1.704	19,6%	4.198	18,6%
	61-70	3.241	23,3%	1.997	22,9%	5.238	23,2%
	71-80	3.528	**25,4%**	2.294	**26,4%**	5.822	**25,7%**
	81-90	2.356	16,9%	1.290	14,8%	3.646	16,1%
	≥ 91	260	1,9%	105	1,2%	365	1,6%
	Gesamt	13.916	100,0%	8.704	100,0%	22.620	100,0%
Systemische Corticosteroide als Kombinationstherapie	1-10	16	0,1%	15	0,2%	31	0,2%
	11-20	157	1,3%	123	1,7%	280	1,4%
	21-30	554	4,4%	378	5,3%	932	4,7%
	31-40	829	6,6%	518	7,3%	1.347	6,9%
	41-50	1.361	10,9%	853	12,0%	2.214	11,3%
	51-60	2.786	22,2%	1.803	**25,4%**	4.589	23,4%
	61-70	3.117	**24,9%**	1.600	22,5%	4.717	**24,0%**
	71-80	2.618	20,9%	1.344	18,9%	3.962	20,2%
	81-90	1.042	8,3%	464	6,5%	1.506	7,7%

Therapiegruppe	Altersgruppen	Weiblich		Männlich		Gesamt	
		n	%	n	%	n	%
	≥ 91	42	0,3%	13	0,2%	55	0,3%
	Gesamt	12.522	100,0%	7.111	100,0%	19.633	100,0%
NSAR – nichtsteroidales Antirheumatikum	1-10	115	0,6%	99	0,7%	214	0,6%
	11-20	235	1,2%	156	1,0%	391	1,1%
	21-30	619	3,1%	435	2,9%	1.054	3,0%
	31-40	1.219	6,2%	1.084	7,2%	2.303	6,6%
	41-50	2.136	10,8%	1.891	12,6%	4.027	11,6%
	51-60	4.181	21,1%	3.699	**24,7%**	7.880	22,7%
	61-70	4.915	**24,8%**	3.537	23,6%	8.452	**24,3%**
	71-80	4.150	20,9%	2.771	18,5%	6.921	19,9%
	81-90	2.017	10,2%	1.210	8,1%	3.227	9,3%
	≥ 91	227	1,1%	85	0,6%	312	0,9%
	Gesamt	19.814	100,0%	14.967	100,0%	34.781	100,0%
Coxibe (COX-2-Hemmer)	1-10	0	0,0%	0	0,0%	0	0,0%
	11-20	9	0,5%	<5	*	11	0,4%
	21-30	44	2,3%	35	3,0%	79	2,6%
	31-40	97	5,2%	101	8,8%	198	6,5%
	41-50	190	10,1%	138	12,0%	328	10,8%
	51-60	415	22,0%	302	**26,2%**	717	**23,6%**
	61-70	447	**23,7%**	268	23,3%	715	23,6%
	71-80	428	22,7%	197	17,1%	625	20,6%
	81-90	216	11,5%	103	8,9%	319	10,5%
	≥ 91	37	2,0%	5	0,4%	42	1,4%
	Gesamt	1.883	100,0%	1.151	100,0%	3.034	100,0%
Nicht-steroidale Antipsoriatika zur dermalen Anwendung als Monotherapie	1-10	16	0,3%	11	0,2%	27	0,2%
	11-20	109	2,3%	108	1,7%	217	1,9%
	21-30	341	7,1%	377	6,0%	718	6,5%
	31-40	518	10,8%	744	11,8%	1.262	11,3%
	41-50	634	13,2%	1.022	16,2%	1.656	14,9%
	51-60	1.121	**23,3%**	1.570	**24,8%**	2.691	**24,2%**
	61-70	1.095	22,8%	1.410	22,3%	2.505	22,5%
	71-80	691	14,4%	825	13,0%	1.516	13,6%
	81-90	253	5,3%	246	3,9%	499	4,5%
	≥ 91	28	0,6%	12	0,2%	40	0,4%
	Gesamt	4.806	100,0%	6.325	100,0%	11.131	100,0%
Nicht-steroidale Antipsoriatika zur dermalen Anwendung als Kombinationstherapie	1-10	<5	*	0	0,0%	<5	*
	11-20	19	2,0%	14	1,0%	33	1,4%
	21-30	55	5,9%	93	6,9%	148	6,5%
	31-40	100	10,8%	187	13,8%	287	12,6%
	41-50	144	15,5%	311	23,0%	455	19,9%
	51-60	273	**29,4%**	373	**27,6%**	646	**28,3%**
	61-70	227	24,4%	234	17,3%	461	20,2%
	71-80	92	9,9%	116	8,6%	208	9,1%
	81-90	15	1,6%	23	1,7%	38	1,7%

Therapiegruppe	Altersgruppen	Weiblich		Männlich		Gesamt	
		n	%	n	%	n	%
	≥ 91	<5	*	<5	*	<5	*
	Gesamt	930	100,0%	1.352	100,0%	2.282	100,0%

* Werte können aus Datenschutzgründen nicht ausgewiesen werden

Tab. S4: Demografie der Therapieformen – Morbus Crohn

Therapiegruppe	Altersgruppen	Weiblich		Männlich		Gesamt	
		n	%	n	%	n	%
Biologika/JAKi	1-10	<5	*	5	0,4%	8	0,3%
	11-20	90	7,2%	109	8,9%	199	8,0%
	21-30	271	**21,6%**	267	21,7%	538	21,6%
	31-40	267	21,3%	286	**23,3%**	553	**22,3%**
	41-50	208	16,6%	214	17,4%	422	17,0%
	51-60	261	20,8%	215	17,5%	476	19,2%
	61-70	121	9,6%	95	7,7%	216	8,7%
	71-80	32	2,5%	36	2,9%	68	2,7%
	81-90	<5	*	<5	*	5	0,2%
	≥ 91	0	0,0%	0	0,0%	0	0,0%
	Gesamt	1.256	100,0%	1.229	100,0%	2.485	100,0%
csDMARDs & klassische systemische Therapien	1-10	11	0,4%	10	0,5%	21	0,5%
	11-20	95	3,8%	112	5,2%	207	4,4%
	21-30	264	10,5%	244	11,4%	508	10,9%
	31-40	338	13,4%	322	15,1%	660	14,2%
	41-50	400	15,9%	359	16,8%	759	16,3%
	51-60	618	**24,5%**	473	**22,2%**	1.091	**23,4%**
	61-70	445	17,7%	343	16,1%	788	16,9%
	71-80	240	9,5%	202	9,5%	442	9,5%
	81-90	95	3,8%	70	3,3%	165	3,5%
	≥ 91	14	0,6%	0	0,0%	14	0,3%
	Gesamt	2.520	100,0%	2.135	100,0%	4.655	100,0%
Systemische Corticosteroide als Monotherapie	1-10	<5	*	<5	*	<5	*
	11-20	6	0,8%	6	1,4%	12	1,1%
	21-30	51	7,2%	40	9,2%	91	8,0%
	31-40	93	13,2%	52	11,9%	145	12,7%
	41-50	98	13,9%	75	17,2%	173	15,1%
	51-60	188	**26,6%**	110	**25,2%**	298	**26,1%**
	61-70	139	19,7%	68	15,6%	207	18,1%
	71-80	88	12,5%	56	12,8%	144	12,6%
	81-90	38	5,4%	26	6,0%	64	5,6%
	≥ 91	<5	*	<5	*	6	0,5%
	Gesamt	706	100,0%	436	100,0%	1.142	100,0%
Systemische Corticosteroide als Kombinationstherapie	1-10	7	0,6%	7	0,8%	14	0,7%
	11-20	71	6,2%	65	7,0%	136	6,5%
	21-30	173	15,1%	152	16,3%	325	15,6%
	31-40	166	14,5%	147	15,8%	313	15,1%
	41-50	167	14,6%	139	14,9%	306	14,7%
	51-60	263	**22,9%**	224	**24,0%**	487	**23,4%**
	61-70	184	16,0%	101	10,8%	285	13,7%
	71-80	79	6,9%	71	7,6%	150	7,2%
	81-90	34	3,0%	26	2,8%	60	2,9%

Therapiegruppe	Altersgruppen	Weiblich		Männlich		Gesamt	
		n	%	n	%	n	%
	≥ 91	<5	*	0	0,0%	<5	*
	Gesamt	1.147	100,0%	932	100,0%	2.079	100,0%
NSAR – nichtsteroidales Antirheumatikum	1-10	8	1,0%	11	1,8%	19	1,3%
	11-20	13	1,6%	6	1,0%	19	1,3%
	21-30	57	7,0%	37	6,2%	94	6,7%
	31-40	115	14,1%	86	14,5%	201	14,3%
	41-50	130	16,0%	114	19,2%	244	17,3%
	51-60	199	**24,5%**	158	**26,6%**	357	**25,4%**
	61-70	173	21,3%	115	19,3%	288	20,5%
	71-80	85	10,5%	49	8,2%	134	9,5%
	81-90	30	3,7%	18	3,0%	48	3,4%
	≥ 91	<5	*	<5	*	<5	*
	Gesamt	813	100,0%	595	100,0%	1.408	100,0%
Coxibe (COX-2-Hemmer)	1-10	0	0,0%	0	0,0%	0	0,0%
	11-20	<5	*	0	0,0%	<5	*
	21-30	7	10,6%	<5	*	8	7,6%
	31-40	6	9,1%	<5	*	8	7,6%
	41-50	12	18,2%	7	17,9%	19	18,1%
	51-60	19	**28,8%**	9	23,1%	28	**26,7%**
	61-70	7	10,6%	14	**35,9%**	21	20,0%
	71-80	8	12,1%	<5	*	12	11,4%
	81-90	6	9,1%	<5	*	8	7,6%
	≥ 91	0	0,0%	0	0,0%	0	0,0%
	Gesamt	66	100,0%	39	100,0%	105	100,0%
Nicht-steroidale Antipsoriatika zur dermalen Anwendung als Monotherapie	1-10	0	0,0%	0	0,0%	0	0,0%
	11-20	0	0,0%	0	0,0%	0	0,0%
	21-30	5	13,5%	<5	*	6	9,5%
	31-40	<5	*	5	19,2%	8	12,7%
	41-50	<5	*	<5	*	5	7,9%
	51-60	9	24,3%	5	19,2%	14	22,2%
	61-70	10	**27,0%**	9	**34,6%**	19	**30,2%**
	71-80	8	21,6%	<5	*	10	15,9%
	81-90	0	0,0%	<5	*	<5	*
	≥ 91	0	0,0%	0	0,0%	0	0,0%
	Gesamt	37	100,0%	26	100,0%	63	100,0%
Nicht-steroidale Antipsoriatika zur dermalen Anwendung als Kombinationstherapie	1-10	0	0,0%	0	0,0%	0	0,0%
	11-20	<5	*	<5	*	<5	*
	21-30	7	14,3%	<5	*	9	10,7%
	31-40	<5	*	6	17,1%	10	11,9%
	41-50	11	22,4%	13	**37,1%**	24	**28,6%**
	51-60	13	**26,5%**	<5	*	17	20,2%
	61-70	9	18,4%	7	20,0%	16	19,0%
	71-80	<5	*	<5	*	<5	*
	81-90	0	0,0%	0	0,0%	0	0,0%

Therapiegruppe	Altersgruppen	Weiblich		Männlich		Gesamt	
		n	%	n	%	n	%
	≥ 91	0	0,0%	0	0,0%	0	0,0%
	Gesamt	49	100,0%	35	100,0%	84	100,0%

* Werte können aus Datenschutzgründen nicht ausgewiesen werden

Tab. S5: Demografie der Therapieformen – Colitis ulcerosa

Therapiegruppe	Altersgruppen	Weiblich		Männlich		Gesamt	
		n	%	n	%	n	%
Biologika/JAKi	1-10	<5	*	<5	*	7	0,5%
	11-20	37	5,5%	42	5,4%	79	5,4%
	21-30	130	19,3%	139	17,9%	269	18,5%
	31-40	150	**22,2%**	147	18,9%	297	**20,5%**
	41-50	124	18,4%	145	18,7%	269	18,5%
	51-60	129	19,1%	162	**20,8%**	291	20,0%
	61-70	72	10,7%	95	12,2%	167	11,5%
	71-80	26	3,9%	37	4,8%	63	4,3%
	81-90	<5	*	6	0,8%	10	0,7%
	≥ 91	0	0,0%	0	0,0%	0	0,0%
	Gesamt	675	100,0%	777	100,0%	1.452	100,0%
csDMARDs & klassische systemische Therapien	1-10	15	0,4%	16	0,4%	31	0,4%
	11-20	122	3,2%	111	2,6%	233	2,8%
	21-30	356	9,2%	370	8,5%	726	8,9%
	31-40	612	15,9%	607	14,0%	1.219	14,9%
	41-50	646	16,8%	724	16,7%	1.370	16,7%
	51-60	820	**21,3%**	995	**22,9%**	1.815	**22,2%**
	61-70	627	16,3%	782	18,0%	1.409	17,2%
	71-80	394	10,2%	517	11,9%	911	11,1%
	81-90	238	6,2%	212	4,9%	450	5,5%
	≥ 91	19	0,5%	10	0,2%	29	0,4%
	Gesamt	3.849	100,0%	4.344	100,0%	8.193	100,0%
Systemische Corticosteroide als Monotherapie	1-10	<5	*	<5	*	<5	*
	11-20	0	0,0%	<5	*	<5	*
	21-30	16	3,2%	21	4,5%	37	3,8%
	31-40	35	7,1%	31	6,6%	66	6,8%
	41-50	56	11,3%	54	11,5%	110	11,4%
	51-60	114	23,0%	122	**26,0%**	236	**24,5%**
	61-70	117	**23,6%**	83	17,7%	200	20,7%
	71-80	102	20,6%	105	22,3%	207	21,5%
	81-90	48	9,7%	49	10,4%	97	10,1%
	≥ 91	6	1,2%	<5	*	8	0,8%
	Gesamt	495	100,0%	470	100,0%	965	100,0%
Systemische Corticosteroide als Kombinationstherapie	1-10	7	0,6%	10	0,7%	17	0,7%
	11-20	59	4,9%	56	4,0%	115	4,4%
	21-30	150	12,5%	168	12,0%	318	12,2%
	31-40	174	14,5%	188	13,4%	362	13,9%
	41-50	192	16,0%	218	15,6%	410	15,8%
	51-60	247	**20,6%**	329	**23,5%**	576	**22,1%**
	61-70	185	15,4%	228	16,3%	413	15,9%
	71-80	109	9,1%	140	10,0%	249	9,6%
	81-90	74	6,2%	62	4,4%	136	5,2%

Therapiegruppe	Altersgruppen	Weiblich		Männlich		Gesamt	
		n	%	n	%	n	%
	≥ 91	<5	*	<5	*	6	0,2%
	Gesamt	1.201	100,0%	1.401	100,0%	2.602	100,0%
NSAR – nichtsteroidales Antirheumatikum	1-10	5	0,6%	7	1,0%	12	0,8%
	11-20	11	1,4%	6	0,9%	17	1,2%
	21-30	28	3,6%	30	4,3%	58	3,9%
	31-40	52	6,7%	63	9,1%	115	7,8%
	41-50	114	14,6%	100	14,5%	214	14,5%
	51-60	181	**23,2%**	187	**27,0%**	368	**25,0%**
	61-70	180	23,1%	155	22,4%	335	22,8%
	71-80	133	17,1%	100	14,5%	233	15,8%
	81-90	69	8,8%	42	6,1%	111	7,5%
	≥ 91	7	0,9%	<5	*	9	0,6%
	Gesamt	780	100,0%	692	100,0%	1.472	100,0%
Coxibe (COX-2-Hemmer)	1-10	0	0,0%	0	0,0%	0	0,0%
	11-20	0	0,0%	0	0,0%	0	0,0%
	21-30	<5	*	<5	*	<5	*
	31-40	9	11,7%	<5	*	13	9,8%
	41-50	11	14,3%	6	10,9%	17	12,9%
	51-60	18	**23,4%**	23	**41,8%**	41	**31,1%**
	61-70	10	13,0%	6	10,9%	16	12,1%
	71-80	16	20,8%	8	14,5%	24	18,2%
	81-90	12	15,6%	6	10,9%	18	13,6%
	≥ 91	0	0,0%	0	0,0%	0	0,0%
	Gesamt	77	100,0%	55	100,0%	132	100,0%
Nicht-steroidale Antipsoriatika zur dermalen Anwendung als Monotherapie	1-10	0	0,0%	0	0,0%	0	0,0%
	11-20	0	0,0%	0	0,0%	0	0,0%
	21-30	<5	*	<5	*	<5	*
	31-40	<5	*	<5	*	<5	*
	41-50	6	20,0%	5	23,8%	11	21,6%
	51-60	7	**23,3%**	5	23,8%	12	23,5%
	61-70	6	20,0%	7	**33,3%**	13	**25,5%**
	71-80	7	**23,3%**	<5	*	9	17,6%
	81-90	<5	*	0	0,0%	<5	*
	≥ 91	<5	*	0	0,0%	<5	*
	Gesamt	30	100,0%	21	100,0%	51	100,0%
Nicht-steroidale Antipsoriatika zur dermalen Anwendung als Kombinationstherapie	1-10	0	0,0%	0	0,0%	0	0,0%
	11-20	<5	*	<5	*	<5	*
	21-30	<5	*	<5	*	8	10,1%
	31-40	8	18,6%	6	16,7%	14	17,7%
	41-50	<5	*	6	16,7%	10	12,7%
	51-60	9	20,9%	9	**25,0%**	18	**22,8%**
	61-70	12	**27,9%**	6	16,7%	18	**22,8%**
	71-80	<5	*	<5	*	7	8,9%
	81-90	<5	*	0	0,0%	<5	*

Therapiegruppe	Altersgruppen	Weiblich		Männlich		Gesamt	
		n	%	n	%	n	%
	≥ 91	0	0,0%	0	0,0%	0	0,0%
	Gesamt	43	100,0%	36	100,0%	79	100,0%

* Werte können aus Datenschutzgründen nicht ausgewiesen werden

Tab. S6: Demografie der Therapieformen – Psoriasis

Therapiegruppe	Altersgruppen	Weiblich		Männlich		Gesamt	
		n	%	n	%	n	%
Biologika/JAKi	1-10	<5	*	<5	*	5	0,2%
	11-20	36	2,5%	13	0,7%	49	1,5%
	21-30	89	6,2%	107	5,6%	196	5,9%
	31-40	181	12,6%	303	16,0%	484	14,5%
	41-50	231	16,1%	450	23,8%	681	20,5%
	51-60	413	**28,8%**	578	**30,5%**	991	**29,8%**
	61-70	329	22,9%	317	16,7%	646	19,4%
	71-80	128	8,9%	103	5,4%	231	6,9%
	81-90	23	1,6%	22	1,2%	45	1,4%
	≥ 91	<5	*	0	0,0%	<5	*
	Gesamt	1.435	100,0%	1.894	100,0%	3.329	100,0%
csDMARDs & klassische systemische Therapien	1-10	<5	*	<5	*	<5	*
	11-20	12	0,4%	20	0,6%	32	0,5%
	21-30	99	3,0%	132	4,1%	231	3,5%
	31-40	221	6,6%	288	9,0%	509	7,8%
	41-50	420	12,6%	524	16,3%	944	14,4%
	51-60	891	26,8%	956	**29,8%**	1.847	**28,2%**
	61-70	972	**29,2%**	731	22,8%	1.703	26,0%
	71-80	542	16,3%	449	14,0%	991	15,2%
	81-90	153	4,6%	102	3,2%	255	3,9%
	≥ 91	15	0,5%	10	0,3%	25	0,4%
	Gesamt	3.328	100,0%	3.213	100,0%	6.541	100,0%
Systemische Corticosteroide als Monotherapie	1-10	<5	*	10	0,3%	12	0,2%
	11-20	24	0,6%	16	0,5%	40	0,6%
	21-30	74	2,0%	62	1,8%	136	1,9%
	31-40	204	5,5%	152	4,4%	356	5,0%
	41-50	333	9,0%	360	10,4%	693	9,7%
	51-60	796	21,6%	791	22,8%	1.587	22,2%
	61-70	988	**26,8%**	909	**26,2%**	1.897	**26,5%**
	71-80	774	21,0%	771	22,2%	1.545	21,6%
	81-90	439	11,9%	362	10,4%	801	11,2%
	≥ 91	59	1,6%	33	1,0%	92	1,3%
	Gesamt	3.693	100,0%	3.466	100,0%	7.159	100,0%
Systemische Corticosteroide als Kombinationstherapie	1-10	<5	*	0	0,0%	<5	*
	11-20	<5	*	6	0,6%	10	0,4%
	21-30	33	2,4%	28	2,6%	61	2,5%
	31-40	85	6,2%	68	6,3%	153	6,2%
	41-50	173	12,5%	160	14,8%	333	13,5%
	51-60	375	27,2%	330	**30,6%**	705	**28,7%**
	61-70	383	**27,8%**	257	23,8%	640	26,0%
	71-80	247	17,9%	185	17,1%	432	17,6%
	81-90	73	5,3%	43	4,0%	116	4,7%

Therapiegruppe	Altersgruppen	Weiblich		Männlich		Gesamt	
		n	%	n	%	n	%
	≥ 91	6	0,4%	<5	*	9	0,4%
	Gesamt	1.380	100,0%	1.080	100,0%	2.460	100,0%
NSAR – nichtsteroidales Antirheumatikum	1-10	56	0,7%	32	0,4%	88	0,5%
	11-20	108	1,3%	83	1,0%	191	1,2%
	21-30	291	3,6%	256	3,1%	547	3,3%
	31-40	592	7,3%	663	8,0%	1.255	7,7%
	41-50	1.004	12,4%	1.122	13,6%	2.126	13,0%
	51-60	1.897	23,5%	2.190	**26,5%**	4.087	25,0%
	61-70	2.070	**25,6%**	2.057	24,9%	4.127	**25,2%**
	71-80	1.393	17,2%	1.306	15,8%	2.699	16,5%
	81-90	609	7,5%	532	6,4%	1.141	7,0%
	≥ 91	62	0,8%	28	0,3%	90	0,6%
	Gesamt	8.082	100,0%	8.269	100,0%	16.351	100,0%
Coxibe (COX-2-Hemmer)	1-10	0	0,0%	0	0,0%	0	0,0%
	11-20	<5	*	<5	*	<5	*
	21-30	8	1,4%	5	1,1%	13	1,2%
	31-40	30	5,1%	29	6,2%	59	5,6%
	41-50	46	7,8%	53	11,4%	99	9,4%
	51-60	139	23,7%	131	**28,1%**	270	25,6%
	61-70	158	**26,9%**	123	26,4%	281	**26,7%**
	71-80	127	21,6%	80	17,2%	207	19,7%
	81-90	68	11,6%	41	8,8%	109	10,4%
	≥ 91	8	1,4%	<5	*	11	1,0%
	Gesamt	587	100,0%	466	100,0%	1.053	100,0%
Nicht-steroidale Antipsoriatika zur dermalen Anwendung als Monotherapie	1-10	16	0,3%	11	0,2%	27	0,2%
	11-20	108	2,3%	106	1,7%	214	2,0%
	21-30	338	7,2%	372	6,0%	710	6,5%
	31-40	514	10,9%	738	11,8%	1.252	11,4%
	41-50	622	13,2%	1.013	16,2%	1.635	14,9%
	51-60	1.099	**23,3%**	1.550	**24,9%**	2.649	**24,2%**
	61-70	1.075	22,8%	1.389	22,3%	2.464	22,5%
	71-80	663	14,1%	807	12,9%	1.470	13,4%
	81-90	246	5,2%	238	3,8%	484	4,4%
	≥ 91	27	0,6%	12	0,2%	39	0,4%
	Gesamt	4.708	100,0%	6.236	100,0%	10.944	100,0%
Nicht-steroidale Antipsoriatika zur dermalen Anwendung als Kombinationstherapie	1-10	<5	*	0	0,0%	<5	*
	11-20	17	1,9%	13	1,0%	30	1,4%
	21-30	49	5,5%	88	6,7%	137	6,2%
	31-40	98	11,1%	183	14,0%	281	12,8%
	41-50	139	15,7%	303	23,1%	442	20,2%
	51-60	260	**29,4%**	362	**27,7%**	622	**28,4%**
	61-70	216	24,4%	226	17,3%	442	20,2%
	71-80	87	9,8%	111	8,5%	198	9,0%
	81-90	14	1,6%	22	1,7%	36	1,6%

Therapiegruppe	Altersgruppen	Weiblich		Männlich		Gesamt	
		n	%	n	%	n	%
	≥ 91	<5	*	<5	*	<5	*
	Gesamt	884	100,0%	1.309	100,0%	2.193	100,0%

* Werte können aus Datenschutzgründen nicht ausgewiesen werden

Tab. S7: Demografie der Therapieformen – Psoriasis Arthritis

Therapiegruppe	Altersgruppen	Weiblich		Männlich		Gesamt	
		n	%	n	%	n	%
Biologika/JAKi	1-10	<5	*	0	0,0%	<5	*
	11-20	18	1,6%	7	0,6%	25	1,1%
	21-30	61	5,6%	48	4,2%	109	4,9%
	31-40	125	11,5%	152	13,3%	277	12,4%
	41-50	192	17,6%	275	24,0%	467	20,9%
	51-60	354	**32,4%**	390	**34,1%**	744	**33,3%**
	61-70	246	22,5%	207	18,1%	453	20,3%
	71-80	84	7,7%	59	5,2%	143	6,4%
	81-90	8	0,7%	6	0,5%	14	0,6%
	≥ 91	<5	*	0	0,0%	<5	*
	Gesamt	1.091	100,0%	1.144	100,0%	2.235	100,0%
csDMARDs & klassische systemische Therapien	1-10	0	0,0%	0	0,0%	0	0,0%
	11-20	5	0,2%	6	0,4%	11	0,3%
	21-30	51	2,4%	38	2,3%	89	2,4%
	31-40	111	5,3%	98	6,0%	209	5,6%
	41-50	283	13,4%	255	15,7%	538	14,4%
	51-60	599	28,4%	537	**33,0%**	1.136	**30,4%**
	61-70	630	**29,9%**	423	26,0%	1.053	28,2%
	71-80	329	15,6%	229	14,1%	558	14,9%
	81-90	94	4,5%	42	2,6%	136	3,6%
	≥ 91	<5	*	0	0,0%	<5	*
	Gesamt	2.106	100,0%	1.628	100,0%	3.734	100,0%
Systemische Corticosteroide als Monotherapie	1-10	<5	*	0	0,0%	<5	*
	11-20	<5	*	<5	*	<5	*
	21-30	12	1,8%	9	1,9%	21	1,8%
	31-40	41	6,0%	15	3,1%	56	4,8%
	41-50	68	10,0%	45	9,4%	113	9,7%
	51-60	178	26,1%	125	**26,2%**	303	26,1%
	61-70	184	**27,0%**	124	25,9%	308	**26,6%**
	71-80	132	19,4%	103	21,5%	235	20,3%
	81-90	61	9,0%	53	11,1%	114	9,8%
	≥ 91	<5	*	<5	*	5	0,4%
	Gesamt	681	100,0%	478	100,0%	1.159	100,0%
Systemische Corticosteroide als Kombinationstherapie	1-10	0	0,0%	0	0,0%	0	0,0%
	11-20	<5	*	<5	*	7	0,3%
	21-30	36	2,9%	23	2,9%	59	2,9%
	31-40	80	6,5%	56	7,0%	136	6,7%
	41-50	166	13,6%	137	17,0%	303	14,9%
	51-60	368	**30,0%**	258	**32,0%**	626	**30,8%**
	61-70	329	26,9%	201	25,0%	530	26,1%
	71-80	180	14,7%	106	13,2%	286	14,1%
	81-90	60	4,9%	21	2,6%	81	4,0%

Therapiegruppe	Altersgruppen	Weiblich		Männlich		Gesamt	
		n	%	n	%	n	%
	≥ 91	<5	*	0	0,0%	<5	*
	Gesamt	1.225	100,0%	805	100,0%	2.030	100,0%
NSAR – nichtsteroidales Antirheumatikum	1-10	<5	*	<5	*	6	0,4%
	11-20	15	1,7%	<5	*	18	1,2%
	21-30	20	2,3%	10	1,7%	30	2,0%
	31-40	53	6,0%	33	5,6%	86	5,8%
	41-50	120	13,6%	102	17,3%	222	15,1%
	51-60	243	27,6%	173	**29,3%**	416	**28,3%**
	61-70	249	**28,2%**	163	27,6%	412	28,0%
	71-80	138	15,6%	79	13,4%	217	14,7%
	81-90	36	4,1%	21	3,6%	57	3,9%
	≥ 91	5	0,6%	<5	*	8	0,5%
	Gesamt	882	100,0%	590	100,0%	1.472	100,0%
Coxibe (COX-2-Hemmer)	1-10	0	0,0%	0	0,0%	0	0,0%
	11-20	<5	*	0	0,0%	<5	*
	21-30	<5	*	0	0,0%	<5	*
	31-40	11	7,3%	9	13,0%	20	9,1%
	41-50	23	15,3%	<5	*	26	11,9%
	51-60	42	28,0%	25	**36,2%**	67	**30,6%**
	61-70	45	**30,0%**	18	26,1%	63	28,8%
	71-80	15	10,0%	9	13,0%	24	11,0%
	81-90	9	6,0%	<5	*	13	5,9%
	≥ 91	<5	*	<5	*	<5	*
	Gesamt	150	100,0%	69	100,0%	219	100,0%
Nicht-steroidale Antipsoriatika zur dermalen Anwendung als Monotherapie	1-10	0	0,0%	0	0,0%	0	0,0%
	11-20	5	1,3%	<5	*	6	0,7%
	21-30	10	2,6%	13	2,9%	23	2,7%
	31-40	29	7,5%	35	7,8%	64	7,6%
	41-50	43	11,1%	57	12,7%	100	11,9%
	51-60	100	**25,8%**	128	**28,5%**	228	**27,2%**
	61-70	99	25,5%	122	27,2%	221	26,4%
	71-80	83	21,4%	67	14,9%	150	17,9%
	81-90	18	4,6%	26	5,8%	44	5,3%
	≥ 91	<5	*	0	0,0%	<5	*
	Gesamt	388	100,0%	449	100,0%	837	100,0%
Nicht-steroidale Antipsoriatika zur dermalen Anwendung als Kombinationstherapie	1-10	<5	*	0	0,0%	<5	*
	11-20	<5	*	<5	*	<5	*
	21-30	15	4,1%	13	2,8%	28	3,4%
	31-40	39	10,6%	60	13,0%	99	11,9%
	41-50	58	15,8%	113	24,4%	171	20,6%
	51-60	107	**29,2%**	144	**31,1%**	251	**30,2%**
	61-70	103	28,1%	83	17,9%	186	22,4%
	71-80	33	9,0%	45	9,7%	78	9,4%
	81-90	7	1,9%	<5	*	11	1,3%

Therapiegruppe	Altersgruppen	Weiblich		Männlich		Gesamt	
		n	%	n	%	n	%
	≥ 91	<5	*	0	0,0%	<5	*
	Gesamt	367	100,0%	463	100,0%	830	100,0%

* Werte können aus Datenschutzgründen nicht ausgewiesen werden

Tab. S8: Demografie der Therapieformen – Spondylitis ankylosans

Therapiegruppe	Altersgruppen	Weiblich		Männlich		Gesamt	
		n	%	n	%	n	%
Biologika/JAKi	1-10	0	0,0%	0	0,0%	0	0,0%
	11-20	7	1,1%	6	0,6%	13	0,8%
	21-30	46	7,0%	97	9,1%	143	8,3%
	31-40	122	18,6%	218	20,5%	340	19,8%
	41-50	163	24,9%	280	26,3%	443	25,7%
	51-60	187	**28,5%**	294	**27,6%**	481	**27,9%**
	61-70	97	14,8%	122	11,4%	219	12,7%
	71-80	32	4,9%	44	4,1%	76	4,4%
	81-90	<5	*	5	0,5%	6	0,3%
	≥ 91	0	0,0%	0	0,0%	0	0,0%
	Gesamt	655	100,0%	1.066	100,0%	1.721	100,0%
csDMARDs & klassische systemische Therapien	1-10	0	0,0%	0	0,0%	0	0,0%
	11-20	<5	*	<5	*	<5	*
	21-30	24	4,3%	22	3,7%	46	4,0%
	31-40	43	7,6%	46	7,8%	89	7,7%
	41-50	96	17,1%	91	15,4%	187	16,2%
	51-60	183	**32,5%**	167	**28,3%**	350	**30,4%**
	61-70	139	24,7%	153	25,9%	292	25,3%
	71-80	53	9,4%	88	14,9%	141	12,2%
	81-90	22	3,9%	22	3,7%	44	3,8%
	≥ 91	0	0,0%	0	0,0%	0	0,0%
	Gesamt	563	100,0%	590	100,0%	1.153	100,0%
Systemische Corticosteroide als Monotherapie	1-10	0	0,0%	0	0,0%	0	0,0%
	11-20	<5	*	<5	*	7	0,5%
	21-30	18	3,2%	21	2,9%	39	3,0%
	31-40	34	6,0%	33	4,6%	67	5,2%
	41-50	91	15,9%	75	10,5%	166	12,9%
	51-60	161	**28,2%**	170	**23,9%**	331	**25,8%**
	61-70	132	23,1%	167	23,5%	299	23,3%
	71-80	94	16,5%	165	23,2%	259	20,2%
	81-90	33	5,8%	73	10,3%	106	8,3%
	≥ 91	<5	*	5	0,7%	9	0,7%
	Gesamt	571	100,0%	712	100,0%	1.283	100,0%
Systemische Corticosteroide als Kombinationstherapie	1-10	0	0,0%	0	0,0%	0	0,0%
	11-20	0	0,0%	<5	*	<5	*
	21-30	24	5,3%	23	5,7%	47	5,5%
	31-40	47	10,4%	39	9,6%	86	10,1%
	41-50	91	20,2%	75	18,5%	166	19,4%
	51-60	136	**30,2%**	127	**31,4%**	263	**30,8%**
	61-70	100	22,2%	77	19,0%	177	20,7%
	71-80	40	8,9%	51	12,6%	91	10,6%
	81-90	12	2,7%	12	3,0%	24	2,8%

Therapiegruppe	Altersgruppen	Weiblich		Männlich		Gesamt	
		n	%	n	%	n	%
	≥ 91	0	0,0%	0	0,0%	0	0,0%
	Gesamt	450	100,0%	405	100,0%	855	100,0%
NSAR – nichtsteroidales Antirheumatikum	1-10	0	0,0%	0	0,0%	0	0,0%
	11-20	<5	*	8	0,5%	12	0,4%
	21-30	41	3,8%	52	3,1%	93	3,4%
	31-40	104	9,7%	146	8,7%	250	9,1%
	41-50	184	17,1%	250	14,9%	434	15,8%
	51-60	279	**25,9%**	437	**26,1%**	716	**26,0%**
	61-70	249	23,1%	368	22,0%	617	22,4%
	71-80	146	13,6%	287	17,1%	433	15,7%
	81-90	61	5,7%	118	7,0%	179	6,5%
	≥ 91	9	0,8%	8	0,5%	17	0,6%
	Gesamt	1.077	100,0%	1.674	100,0%	2.751	100,0%
Coxibe (COX-2-Hemmer)	1-10	0	0,0%	0	0,0%	0	0,0%
	11-20	0	0,0%	0	0,0%	0	0,0%
	21-30	18	7,7%	24	7,2%	42	7,4%
	31-40	28	12,0%	60	18,1%	88	15,6%
	41-50	42	18,0%	57	17,2%	99	17,5%
	51-60	67	**28,8%**	89	**26,8%**	156	**27,6%**
	61-70	46	19,7%	58	17,5%	104	18,4%
	71-80	21	9,0%	31	9,3%	52	9,2%
	81-90	10	4,3%	12	3,6%	22	3,9%
	≥ 91	<5	*	<5	*	<5	*
	Gesamt	233	100,0%	332	100,0%	565	100,0%
Nicht-steroidale Antipsoriatika zur dermalen Anwendung als Monotherapie	1-10	0	0,0%	0	0,0%	0	0,0%
	11-20	0	0,0%	0	0,0%	0	0,0%
	21-30	<5	*	<5	*	<5	*
	31-40	<5	*	<5	*	5	6,8%
	41-50	<5	*	6	12,5%	9	12,2%
	51-60	5	19,2%	12	25,0%	17	23,0%
	61-70	8	**30,8%**	13	**27,1%**	21	**28,4%**
	71-80	5	19,2%	9	18,8%	14	18,9%
	81-90	<5	*	<5	*	<5	*
	≥ 91	0	0,0%	0	0,0%	0	0,0%
	Gesamt	26	100,0%	48	100,0%	74	100,0%
Nicht-steroidale Antipsoriatika zur dermalen Anwendung als Kombinationstherapie	1-10	0	0,0%	0	0,0%	0	0,0%
	11-20	0	0,0%	0	0,0%	0	0,0%
	21-30	0	0,0%	<5	*	<5	*
	31-40	<5		7	16,7%	11	18,6%
	41-50	5	**29,4%**	8	19,0%	13	22,0%
	51-60	5	**29,4%**	13	**31,0%**	18	**30,5%**
	61-70	<5	*	7	16,7%	10	16,9%
	71-80	0	0,0%	5	11,9%	5	8,5%
	81-90	0	0,0%	0	0,0%	0	0,0%

Therapiegruppe	Altersgruppen	Weiblich		Männlich		Gesamt	
		n	%	n	%	n	%
	≥ 91	0	0,0%	0	0,0%	0	0,0%
	Gesamt	17	100,0%	42	100,0%	59	100,0%

* Werte können aus Datenschutzgründen nicht ausgewiesen werden

Tab. S9: Demografie der Therapieformen – Rheumatoide Arthritis

Therapiegruppe	Altersgruppen	Weiblich		Männlich		Gesamt	
		n	%	n	%	n	%
Biologika/JAKi	1-10	7	0,2%	<5	*	8	0,1%
	11-20	52	1,4%	16	0,9%	68	1,2%
	21-30	146	3,8%	55	3,1%	201	3,6%
	31-40	277	7,2%	123	7,0%	400	7,2%
	41-50	491	12,8%	257	14,7%	748	13,4%
	51-60	1.001	26,1%	584	**33,4%**	1.585	**28,4%**
	61-70	1.040	**27,1%**	428	24,5%	1.468	26,3%
	71-80	668	17,4%	227	13,0%	895	16,0%
	81-90	147	3,8%	58	3,3%	205	3,7%
	≥ 91	<5	*	<5	*	<5	*
	Gesamt	3.831	100,0%	1.750	100,0%	5.581	100,0%
csDMARDs & klassische systemische Therapien	1-10	11	0,1%	<5	*	14	0,1%
	11-20	24	0,2%	14	0,3%	38	0,2%
	21-30	194	1,7%	45	0,8%	239	1,4%
	31-40	434	3,7%	144	2,7%	578	3,4%
	41-50	1.053	9,0%	404	7,6%	1.457	8,5%
	51-60	2.497	21,3%	1.259	23,6%	3.756	22,0%
	61-70	3.296	**28,1%**	1.522	**28,5%**	4.818	**28,2%**
	71-80	2.906	24,8%	1.431	26,8%	4.337	25,4%
	81-90	1.256	10,7%	502	9,4%	1.758	10,3%
	≥ 91	70	0,6%	21	0,4%	91	0,5%
	Gesamt	11.741	100,0%	5.345	100,0%	17.086	100,0%
Systemische Corticosteroide als Monotherapie	1-10	<5	*	<5	*	<5	*
	11-20	7	0,1%	<5	*	9	0,1%
	21-30	58	0,9%	19	0,6%	77	0,8%
	31-40	159	2,4%	48	1,5%	207	2,1%
	41-50	349	5,2%	146	4,7%	495	5,0%
	51-60	999	14,9%	483	15,5%	1.482	15,1%
	61-70	1.489	22,2%	646	20,8%	2.135	21,7%
	71-80	1.957	**29,1%**	1.029	**33,1%**	2.986	**30,4%**
	81-90	1.529	22,7%	678	21,8%	2.207	22,4%
	≥ 91	173	2,6%	60	1,9%	233	2,4%
	Gesamt	6.721	100,0%	3.112	100,0%	9.833	100,0%
Systemische Corticosteroide als Kombinationstherapie	1-10	<5	*	<5	*	<5	*
	11-20	25	0,3%	8	0,2%	33	0,3%
	21-30	176	2,0%	39	1,0%	215	1,7%
	31-40	363	4,0%	105	2,6%	468	3,6%
	41-50	813	9,1%	346	8,6%	1.159	8,9%
	51-60	1.947	21,7%	1.015	25,3%	2.962	22,8%
	61-70	2.459	**27,4%**	1.113	**27,8%**	3.572	**27,5%**
	71-80	2.255	25,1%	1.012	25,3%	3.267	25,2%
	81-90	903	10,1%	354	8,8%	1.257	9,7%

Therapiegruppe	Altersgruppen	Weiblich		Männlich		Gesamt	
		n	%	n	%	n	%
	≥ 91	32	0,4%	12	0,3%	44	0,3%
	Gesamt	8.976	100,0%	4.005	100,0%	12.981	100,0%
NSAR – nichtsteroidales Antirheumatikum	1-10	9	0,2%	<5	*	12	0,2%
	11-20	16	0,3%	19	0,9%	35	0,5%
	21-30	89	1,6%	28	1,3%	117	1,5%
	31-40	173	3,2%	82	3,7%	255	3,3%
	41-50	407	7,5%	199	8,9%	606	7,9%
	51-60	1.076	19,8%	537	24,1%	1.613	21,0%
	61-70	1.424	**26,2%**	542	24,3%	1.966	**25,6%**
	71-80	1.402	25,8%	546	**24,5%**	1.948	25,4%
	81-90	745	13,7%	250	11,2%	995	13,0%
	≥ 91	98	1,8%	25	1,1%	123	1,6%
	Gesamt	5.439	100,0%	2.231	100,0%	7.670	100,0%
Coxibe (COX-2-Hemmer)	1-10	0	0,0%	0	0,0%	0	0,0%
	11-20	0	0,0%	<5	*	<5	*
	21-30	9	1,3%	<5	*	12	1,3%
	31-40	23	3,4%	6	2,5%	29	3,2%
	41-50	66	9,7%	19	8,0%	85	9,3%
	51-60	134	19,7%	45	19,0%	179	19,5%
	61-70	164	24,2%	68	**28,7%**	232	25,3%
	71-80	169	**24,9%**	64	27,0%	233	**25,4%**
	81-90	96	14,1%	30	12,7%	126	13,8%
	≥ 91	18	2,7%	<5	*	19	2,1%
	Gesamt	679	100,0%	237	100,0%	916	100,0%
Nicht-steroidale Antipsoriatika zur dermalen Anwendung als Monotherapie	1-10	0	0,0%	0	0,0%	0	0,0%
	11-20	<5	*	0	0,0%	<5	*
	21-30	<5	*	<5	*	<5	*
	31-40	<5	*	9	6,7%	10	3,4%
	41-50	12	7,5%	12	8,9%	24	8,1%
	51-60	29	18,0%	23	17,0%	52	17,6%
	61-70	55	**34,2%**	39	**28,9%**	94	**31,8%**
	71-80	41	25,5%	35	25,9%	76	25,7%
	81-90	17	10,6%	15	11,1%	32	10,8%
	≥ 91	<5	*	<5	*	<5	*
	Gesamt	161	100,0%	135	100,0%	296	100,0%
Nicht-steroidale Antipsoriatika zur dermalen Anwendung als Kombinationstherapie	1-10	<5	*	0	0,0%	<5	*
	11-20	<5	*	0	0,0%	<5	*
	21-30	<5	*	<5	*	<5	*
	31-40	10	6,8%	8	6,7%	18	6,7%
	41-50	19	12,9%	16	13,3%	35	13,1%
	51-60	41	27,9%	35	29,2%	76	28,5%
	61-70	51	**34,7%**	37	**30,8%**	88	**33,0%**
	71-80	20	13,6%	20	16,7%	40	15,0%
	81-90	<5	*	<5	*	<5	*

Therapiegruppe	Altersgruppen	Weiblich		Männlich		Gesamt	
		n	%	n	%	n	%
	≥ 91	<5	*	0	0,0%	<5	*
	Gesamt	147	100,0%	120	100,0%	267	100,0%

* Werte können aus Datenschutzgründen nicht ausgewiesen werden

Tab. S10: Demografie der Therapieformen – Kollagenosen

Therapiegruppe	Altersgruppen	Weiblich		Männlich		Gesamt	
		n	%	n	%	n	%
Biologika/JAKi	1-10	<5	*	0	0,0%	<5	*
	11-20	5	1,0%	<5	*	9	1,3%
	21-30	37	7,3%	<5	*	41	6,1%
	31-40	46	9,1%	19	11,7%	65	9,7%
	41-50	60	11,8%	32	19,8%	92	13,7%
	51-60	133	**26,2%**	41	**25,3%**	174	**26,0%**
	61-70	131	25,8%	35	21,6%	166	24,8%
	71-80	79	15,6%	19	11,7%	98	14,6%
	81-90	16	3,1%	8	4,9%	24	3,6%
	≥ 91	0	0,0%	0	0,0%	0	0,0%
	Gesamt	508	100,0%	162	100,0%	670	100,0%
csDMARDs & klassische systemische Therapien	1-10	7	0,2%	5	0,6%	12	0,3%
	11-20	43	1,4%	9	1,1%	52	1,3%
	21-30	140	4,4%	26	3,3%	166	4,2%
	31-40	310	9,8%	55	6,9%	365	9,2%
	41-50	457	14,5%	99	12,4%	556	14,0%
	51-60	756	**23,9%**	197	**24,7%**	953	**24,1%**
	61-70	703	22,2%	172	21,6%	875	22,1%
	71-80	555	17,6%	175	21,9%	730	18,4%
	81-90	180	5,7%	58	7,3%	238	6,0%
	≥ 91	9	0,3%	<5	*	11	0,3%
	Gesamt	3.160	100,0%	798	100,0%	3.958	100,0%
Systemische Corticosteroide als Monotherapie	1-10	14	0,5%	13	1,1%	27	0,6%
	11-20	25	0,8%	<5	*	29	0,7%
	21-30	74	2,5%	26	2,3%	100	2,4%
	31-40	126	4,2%	35	3,1%	161	3,9%
	41-50	238	7,9%	67	5,8%	305	7,3%
	51-60	490	16,2%	152	13,3%	642	15,4%
	61-70	716	23,7%	264	23,0%	980	23,5%
	71-80	851	**28,2%**	376	**32,8%**	1.227	**29,5%**
	81-90	453	15,0%	202	17,6%	655	15,7%
	≥ 91	30	1,0%	7	0,6%	37	0,9%
	Gesamt	3.017	100,0%	1.146	100,0%	4.163	100,0%
Systemische Corticosteroide als Kombinationstherapie	1-10	<5	*	<5	*	7	0,3%
	11-20	22	1,1%	<5	*	25	1,0%
	21-30	98	5,1%	14	2,7%	112	4,6%
	31-40	160	8,4%	37	7,2%	197	8,1%
	41-50	258	13,5%	67	13,0%	325	13,4%
	51-60	463	**24,2%**	130	**25,3%**	593	**24,4%**
	61-70	446	23,3%	111	21,6%	557	22,9%
	71-80	352	18,4%	113	22,0%	465	19,2%
	81-90	109	5,7%	35	6,8%	144	5,9%

Therapiegruppe	Altersgruppen	Weiblich		Männlich		Gesamt	
		n	%	n	%	n	%
	≥ 91	<5	*	<5	*	<5	*
	Gesamt	1.914	100,0%	514	100,0%	2.428	100,0%
NSAR – nichtsteroidales Antirheumatikum	1-10	37	0,8%	45	2,2%	82	1,3%
	11-20	77	1,7%	36	1,8%	113	1,7%
	21-30	128	2,8%	39	1,9%	167	2,6%
	31-40	232	5,2%	66	3,3%	298	4,6%
	41-50	374	8,3%	145	7,2%	519	8,0%
	51-60	721	16,0%	304	15,2%	1.025	15,7%
	61-70	1.052	23,4%	426	21,2%	1.478	22,7%
	71-80	1.203	**26,7%**	611	**30,5%**	1.814	**27,9%**
	81-90	622	13,8%	313	15,6%	935	14,4%
	≥ 91	56	1,2%	21	1,0%	77	1,2%
	Gesamt	4.502	100,0%	2.006	100,0%	6.508	100,0%
Coxibe (COX-2-Hemmer)	1-10	0	0,0%	0	0,0%	0	0,0%
	11-20	<5	*	0	0,0%	<5	*
	21-30	7	1,8%	<5	*	8	1,6%
	31-40	13	3,3%	5	4,6%	18	3,6%
	41-50	33	8,5%	8	7,4%	41	8,2%
	51-60	75	19,2%	27	25,0%	102	20,5%
	61-70	92	23,6%	22	20,4%	114	22,9%
	71-80	111	**28,5%**	28	**25,9%**	139	**27,9%**
	81-90	43	11,0%	17	15,7%	60	12,0%
	≥ 91	12	3,1%	0	0,0%	12	2,4%
	Gesamt	390	100,0%	108	100,0%	498	100,0%
Nicht-steroidale Antipsoriatika zur dermalen Anwendung als Monotherapie	1-10	0	0,0%	0	0,0%	0	0,0%
	11-20	0	0,0%	<5	*	<5	*
	21-30	<5	*	<5	*	5	2,9%
	31-40	<5	*	<5	*	6	3,5%
	41-50	7	6,7%	5	7,4%	12	7,0%
	51-60	21	20,2%	9	13,2%	30	17,4%
	61-70	31	**29,8%**	21	**30,9%**	52	**30,2%**
	71-80	30	28,8%	16	23,5%	46	26,7%
	81-90	10	9,6%	9	13,2%	19	11,0%
	≥ 91	0	0,0%	0	0,0%	0	0,0%
	Gesamt	104	100,0%	68	100,0%	172	100,0%
Nicht-steroidale Antipsoriatika zur dermalen Anwendung als Kombinationstherapie	1-10	<5	*	0	0,0%	<5	*
	11-20	0	0,0%	<5	*	<5	*
	21-30	<5	*	0	0,0%	<5	*
	31-40	<5	*	0	0,0%	<5	*
	41-50	9	22,5%	<5	*	11	20,8%
	51-60	9	22,5%	<5	*	13	24,5%
	61-70	11	**27,5%**	<5	*	15	**28,3%**
	71-80	6	15,0%	<5	*	8	15,1%
	81-90	<5	*	0	0,0%	<5	*

Therapiegruppe	Altersgruppen	Weiblich		Männlich		Gesamt	
		n	%	n	%	n	%
	≥ 91	0	0,0%	0	0,0%	0	0,0%
	Gesamt	40	100,0%	13	100,0%	53	100,0%

* Werte können aus Datenschutzgründen nicht ausgewiesen werden
